# Wireless Acousto‐Piezoelectric Conduit with Aligned Nanofibers for Neural Regeneration

**DOI:** 10.1002/adma.202503343

**Published:** 2025-09-02

**Authors:** Sera Jeon, Dabin Kim, Min‐Young Jo, Chae‐Min Ryu, Daniel Sanghyun Cho, Byung‐Ok Choi, Jae Kwang Kim, Miso Kim, Sang‐Woo Kim

**Affiliations:** ^1^ Department of Materials Science and Engineering Center for Human‐oriented Triboelectric Energy Harvesting Yonsei University Seoul 03722 Republic of Korea; ^2^ Department of Orthopedic Surgery Asan Medical Center University of Ulsan College of Medicine Seoul 05505 Republic of Korea; ^3^ Center for Cell Therapy Asan Medical Center Seoul 05505 Republic of Korea; ^4^ Department of Neurology Samsung Medical Center Sungkyunkwan University School of Medicine Seoul 06351 Republic of Korea; ^5^ Department of Mechanical Engineering Korea Advanced Institute of Science and Technology Daejeon 34141 Republic of Korea

**Keywords:** electrospun nanofibers, nerve guidance conduits, peripheral nerve injuries, piezoelectric polymers, regenerative scaffolds

## Abstract

Peripheral nerve injury (PNI) represents a significant clinical challenge, leading to severe motor and sensory dysfunction, as well as irreversible tissue atrophy. Autograft has been commonly utilized as the clinical gold standard; however, it is limited by donor availability and secondary surgery requirements. Here, an ultrasound‐responsive, highly aligned piezoelectric nanofiber nerve guidance conduit (APNF‐NGC) is introduced for peripheral nerve regeneration. Fabricated from electrospun poly‐*l*‐lactic acid (PLLA) nanofibers, the APNF‐NGC features an anisotropically oriented architecture with shear piezoelectricity, providing both structural support and wireless electrical stimulation. The incorporation of polyethylene glycol (PEG) tailors mechanical properties, increases piezoelectric‐phase crystallinity, and improves the surface hydrophilicity, thereby enhancing both biocompatibility and acousto‐piezoelectric response. Finite element analysis and electrical assessment confirm that ultrasound activation of the APNF‐NGC generates an axially oriented electric field, facilitating directional axon elongation. In vivo studies using an 8‐mm sciatic nerve defect rat model demonstrated that the APNF‐NGC achieved nerve reinnervation comparable to that of autografts, as comprehensively validated by behavioral, motor function, and histological evaluations. This dual‐function platform, combining physical guidance with electrical stimulation, presents a promising strategy for neural tissue engineering, offers a potential breakthrough in treating long‐gap PNIs.

## Graphical abstract

An ultrasound‐driven, highly aligned piezoelectric nanofiber‐based nerve guidance conduit (APNF‐NGC) designed for effective peripheral nerve repair has been demonstrated in this study. Featuring aligned topology, cell‐adhesive surfaces, and an axial electric field, the APNF‐NGC collectively promotes significant nerve regeneration. In vivo experiments validate its effectiveness, revealing nerve repair outcomes comparable to those achieved with autografts.

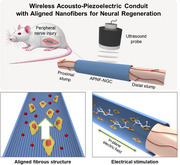

## 1 Introduction

Peripheral nerve injury (PNI) is a significant clinical issue that affects millions of people worldwide, often resulting from trauma, overstretching, fractures, or iatrogenic causes. Nerve defects associated with PNI frequently lead to severe motor and sensory dysfunction as well as irreversible tissue atrophy. While autologous nerve transplantation remains the gold standard for treating peripheral nerve defects, it has several limitations, including restricted donor availability, size mismatches, and the risk of secondary surgical trauma.^[^ ^1^, ^2^ ^]^ In response to these challenges, nerve guidance conduits (NGCs) have emerged as promising alternatives to autografts for bridging nerve gaps and promoting regeneration.^[^ ^3^, ^4^ ^]^ An ideal NGC requires a porous and aligned morphology, biocompatibility, adequate mechanical strength, and adaptability in terms of both size and shape.

Electrical stimulation is known to enhance cellular activities such as proliferation and differentiation, which are critical for promoting nerve growth and repair.^[^ ^5^, ^6^ ^]^Recent implantable systems have leveraged this effect through wireless technologies such as inductive coupling and ultrasound‐driven triboelectric nanogenerators (TENGs) for peripheral nerve regeneration and chronic disease treatment.^[^ ^7^, ^8^, ^9^ ^]^ However, inductive systems are limited by shallow energy transmission depth and require precise alignment between the transmitter and receiver. Implantable TENGs employ multilayered structures and encapsulation to maintain frictional contact and protect internal components, which complicates fabrication and implantation. Additionally, these implantable systems typically equip separated energy generation and stimulation unit using lead wires, resulting in bulky configurations relative to the size of the target nerve. Recently, piezoelectric materials have shown promise in regenerative therapy. Polyvinylidene fluoride (PVDF) and its copolymers have been developed into NGCs and demonstrated effectiveness in promoting Schwann cell growth.^[^ ^10^, ^11^, ^12^ ^]^ However, due to regulations regarding per‐ and polyfluoroalkyl substances (PFAS) and non‐degradability, PVDF copolymers may not be ideal for developing next‐generation NGCs.^[^ ^13^ ^]^ While biopolymers such as collagen, FF nanotubes, and chitosan are known for their biocompatibility, they often fall short in mechanical strength, preventing them from maintaining structural integrity and supporting the necessary cellular activities for effective nerve regeneration during the typical 3–4 months recovery period.^[^ ^14^, ^15^, ^16^ ^]^ Although poly(L‐lactic acid (PLLA) possesses desirable biocompatibility and piezoelectric properties, its hydrophobicity and rigid mechanical characteristics pose challenges to its application.^[^ ^17^, ^18^ ^]^

To address these challenges, we introduce an ultrasound‐driven, highly aligned piezoelectric nanofiber (APNF)‐based NGC (APNF‐NGC) designed specifically for peripheral nerve regeneration. Owing to its unique morphology and piezoelectric properties, the APNF‐NGC provides both structural support and wireless electrical stimulation upon ultrasound activation. We selected ultrasound as the non‐invasive activation method due to its established clinical safety, and deep tissue penetration capability.^[^ ^19^, ^20^, ^21^ ^]^ Using electrospinning, APNF was fabricated from PLLA blended with polyethylene glycol (PEG), resulting in an anisotropically oriented topology. The incorporation of PEG was carefully tailored to modulate the mechanical strength, enhance α‐phase crystallinity, and create a hydrophilic surface, thereby improving both biocompatibility and piezoelectricity. When activated by ultrasound, the APNF‐NGC generates an electric field along the axial direction, as demonstrated through finite element analysis (FEA) and electrical measurements. In vivo experiments in a rat model with an 8 mm sciatic nerve injury further validated the efficacy of APNF‐NGC, showing comparable nerve reinnervation to autografts. Behavioral, motor function and histological assessments comprehensively confirmed accelerated functional recovery and axonal regeneration facilitated by APNF‐NGC. This study offers a promising dual‐function solution for neural tissue engineering, combining physical guidance and electrical stimulation to enhance nerve regeneration, potentially revolutionizing treatment strategies for long‐gap PNIs.

## 2 Results and Discussion

**Figure 1:**
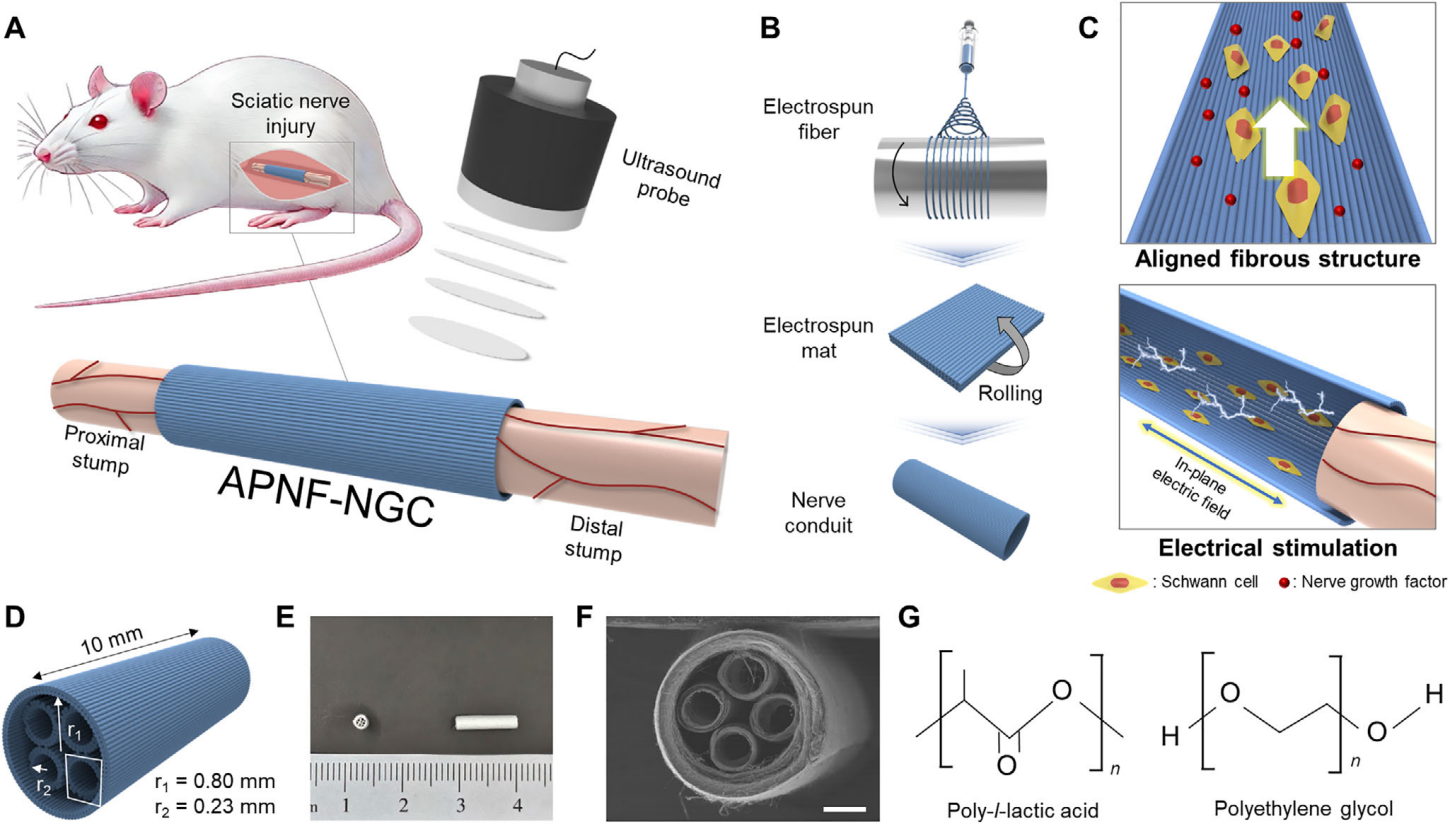
Overview and fabrication process of the APNF‐NGC. A) Schematic diagram of the ultrasound‐driven APNF‐NGC for peripheral nerve regeneration. B) Illustration of the roles of APNF in promoting nerve regeneration. C) Fabrication process of the APNF‐NGC using electrospinning, followed by rolling the mat into a conduit. D) Schematic illustration of the APNF‐NGC design, showing the four‐channel cylindrical structure. E) Photograph and F) SEM image of the APNF‐NGC (Scale bar = 500 µm). G) Molecular structures of APNF materials: PLLA and PEG.

**Figure** **1**A illustrates the overall concept of our APNF‐NGC, which is implanted at the site of peripheral nerve defect, bridging the proximal and distal stumps, while transcutaneous ultrasound is applied to induce piezoelectricity. We employed electrospinning to synthesize highly aligned piezoelectric nanofibers, which were subsequently formed into a conduit by rolling the electrospun mat (Figure 1B; Figure S1, Supporting Information). The APNF‐NGC combines both structural and electrical features to facilitate nerve regeneration (Figure 1C): 1) an aligned fibrous structure that supports cell growth and guides nerve growth factor (NGF) release, and 2) an in‐plane electric field to electrically promote neuroregeneration. Additionally, the fibrous structure of the NGC allows for oxygen permeability through its porosity, which is crucial for nerve regeneration.^[^ ^22^ ^]^ Figure 1D–F presents schematics and images of our APNF‐NGC, comprising a 1.6 mm‐diameter conduit (250 µm in thickness) with four smaller internal channels (0.46 mm in diameter and 70 µm in thickness). The NGC possesses a total length of 10 mm, optimized for the target nerve repair in our in vivo test. The APNF‐NGC was designed with a four‐channel configuration to prevent guidance failure caused by gravitational forces acting on the regenerating nerve and to resist collapse under pressure from surrounding tissues. In addition to enhancing mechanical robustness, this architecture biomimetically mimics the fascicular organization of the rat sciatic nerve, which typically contains 3 to 4 fascicles with diameters of 100–500 µm.^[^ ^23^ ^]^ Each channel in the conduit had a diameter of ≈0.46 mm, closely matching the size of individual nerve fascicles. The channels also offer pathways for cell infiltration, vascular ingrowth, and directional diffusion of neurotrophic factors (e.g., NGF, BDNF), helping guide axon elongation and promote neuronal survival. Figure 1G introduces the material composition of the NGC, which consists of hydrophilic, aligned piezoelectric nanofibers made from a blend of PLLA and PEG. PLLA serves as the primary material due to its biocompatibility, mechanical robustness, and high piezoelectric properties. At the same time, PEG is incorporated to reinforce the flexibility, crystallinity, and hydrophilicity of the PLLA nanofibers.

We prepared PLLA/PEG solutions with weight ratios of 100:0, 80:20, 60:40, 40:60, and 20:80 and processed each into nanofibers via electrospinning. Figure S2 (Supporting Information) shows scanning electron microscopy (SEM) images of the electrospun products for each ratio. When the PEG fraction exceeded 80%, bead formation was observed instead of uniform nanofibers, while 40% and 60% PEG fractions exhibited irregular nanofiber diameters. This outcome is attributed to PEG's lower molecular weight compared to PLLA, which diluted the polymer solution and affected its spinnability. Therefore, we limited the PEG content to 30%, with final ratios of 100:0, 90:10, 80:20, and 70:30, denoted as PLLA, PEG10, PEG20, and PEG30, respectively.

**Figure 2:**
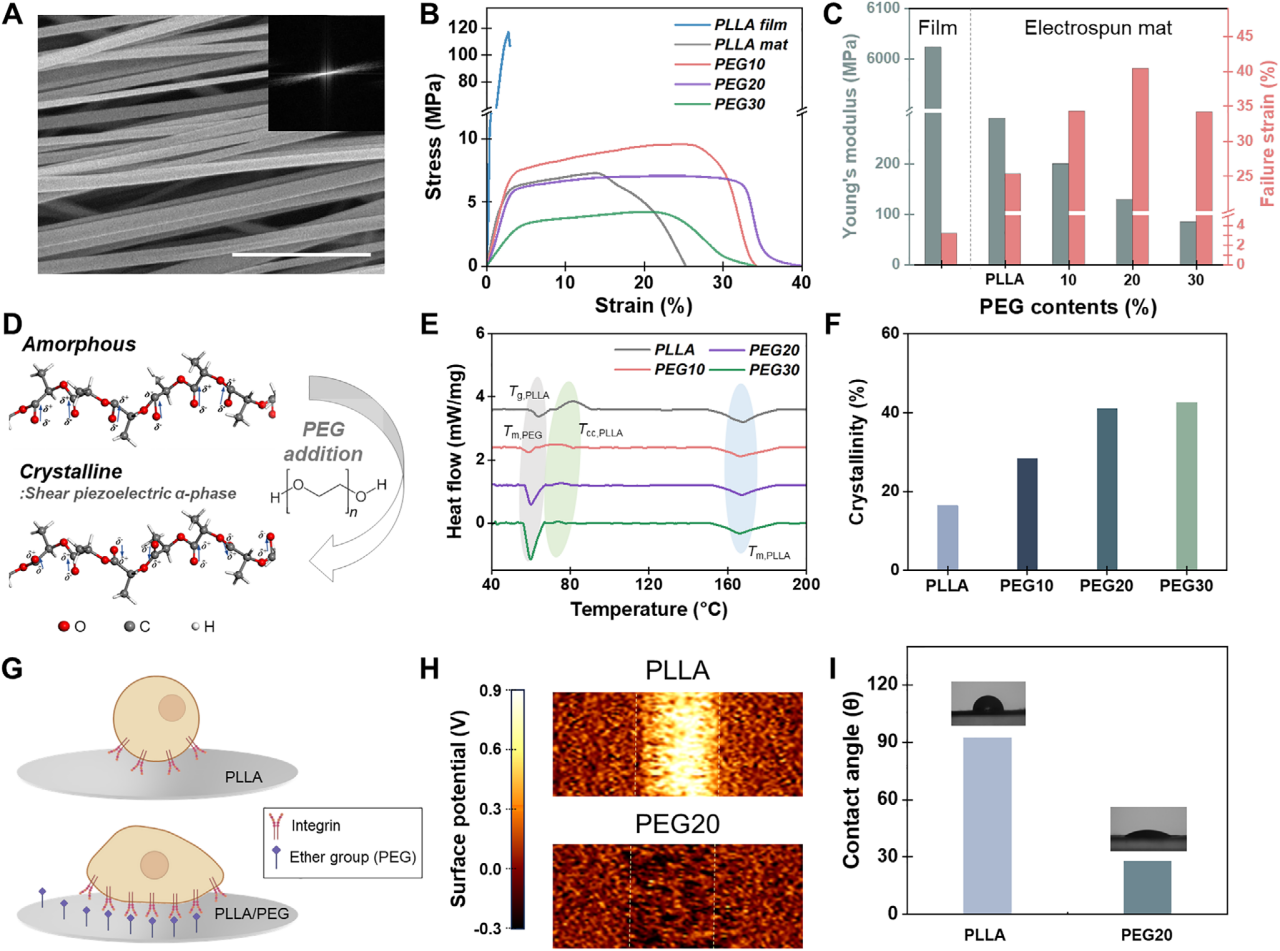
Characterization of electrospun APNF. A) SEM image of electrospun PLLA deposited at a linear speed of 18.4 m s^−1^ and inset image is its FFT to assess the alignment. B) Stress‐strain curve and C) Young's modulus of PLLA film and PLLA and its PEG blend (PLLA/PEG) electrospun mat. D) Effect of PEG blend on PLLA molecular crystallinity. E) DSC thermograms of the PLLA/PEG electrospun mat with different PEG concentrations and F) their α‐phase PLLA content. G) Schematic illustrating the hydrophilicity and surface charge on cell growth. H) Surface potential of PLLA and PEG20 single nanofiber strand. I) Contact angles of PLLA and PEG20 electrospun mat.

Then, anisotropically oriented APNF were produced by controlling the rotational speed of the collector, which mechanically stretched the fibers during deposition onto the spinning drum. To evaluate fiber alignment and diameter distribution, we observed the electrospun PLLA nanofiber mat using SEM (**Figure** **2**A; Figure S3, Supporting Information). Fiber anisotropy became prominent when the rpm exceeded 1000. Further quantitative analysis using the fast Fourier transform (FFT) from an SEM image confirmed that fiber alignment reached its maximum at 2000 rpm, corresponding to a linear speed of 18.8 m s^−1^ (Figure S4, Supporting Information).^[^ ^24^, ^25^ ^]^ The nanofibers, particularly those produced at 2000 rpm, exhibited a diameter distribution of 1.18 ± 0.48 µm, with smooth surfaces (Figure S5, Supporting Information).^[^ ^26^ ^]^ The higher rotational speed of the collector not only enhances the orientation of nanofibers but is also anticipated to improve the crystallinity of PLLA by physically drawing the fiber.^[^ ^27^ ^]^ XRD patterns of random and aligned PLLA nanofibers, fabricated at 100 rpm and 2000 rpm respectively, revealed significantly higher α‐phase crystallinity in the aligned fibers (Figure S6, Supporting Information). This is evidenced by the sharper diffraction peak at 16.3 °, attributed to the molecular stretching induced by deposition on the rapidly rotating collector.

The electrospun APNF serves not only as a porous, highly aligned physical guide for nerve regeneration but also optimizes the mechanical properties to ensure biocompatibility with surrounding tissues. To function effectively in nerve guidance applications, the APNF‐NGC requires sufficient mechanical strength to prevent collapse and maintain structural integrity while supporting guided axon regeneration—particularly in bridging long‐gap nerve defects, including those on the centimeter scale that are frequently encountered in clinical settings.^[^ ^28^, ^29^, ^30^, ^31^ ^]^ Given that neural tissues are inherently soft and pliable, stiffness and ductility are key considerations when designing an ideal NGC.

Therefore, we aimed to evaluate the mechanical compatibility with nerve tissues through Young's modulus and assess whether the NGC can provide sufficient flexibility via failure strain obtained from the stress‐strain curve (Figure 2B).^[^ ^32^ ^]^ PLLA film exhibited Young's modulus of 6.02 GPa, considerably higher than native nerve tissue, which generally is between a few and tens of MPa. By employing electrospinning, even with identical materials, we managed to lower the modulus to 289.86 MPa, and further reductions were observed as the PEG fraction increased (Figure 2C). For optimal structural integrity capable of bridging extensive nerve gaps and effectively guiding axonal growth, we identified PEG20, exhibiting 128.73 MPa, as the ideal PLLA/PEG blend concentration.^[^ ^33^, ^34^, ^35^ ^]^ We also examined APNF to analyze the failure strain, which ensures the NGC can withstand deformation and external stress without rupturing. The film‐type PLLA had a failure strain of 3.17%, but this was markedly enhanced by electrospinning, with PEG20 achieving the highest failure strain (40.35%). The observed reduction in stiffness and increase in ductility result from the synergistic effects of highly aligned nanofibers and PEG‐induced molecular mobility, which together enhance the mechanical compatibility of the scaffold as NGCs.

For our APNF‐NGC, it is necessary to achieve α‐phase PLLA, as it exhibits shear piezoelectricity (*d*_14_) (Figure S7, Supporting Information). Although the electrospinning process yields a highly aligned nanofiber morphology, the molecular structures remain predominantly amorphous because of the extremely strong electric field during electrospinning. The incorporation of PEG facilitates α‐phase crystallization by enhancing the mobility of PLLA chains. Specifically, PEG acts as a plasticizer that reduces intermolecular interactions and increases the free volume, thereby lowering the cold crystallization temperature (*T*_cc_) and promoting more ordered molecular packing (Figure 2D).^[^ ^36^, ^37^, ^38^, ^39^ ^]^ To quantify the crystallinity induced by PEG addition, we investigated the thermal transitions of all PLLA/PEG electrospun mats using differential scanning calorimetry (DSC) scans. The DSC thermograms from the heating cycle are shown in Figure 2E, and crystallinity (*χ* ^PLLA^) was clculated using the following Equation (1):

(1) $$\chi^{PLLA}\left(\%\right)=\frac{\Delta H_{m}-\Delta H_{cc}}{f_{PLLA}\Delta H_{m}^{0}}$$

where *ΔH_m_* is the measured heat of fusion, *ΔH_cc_* is the heat of cold crystalization, *f_PLLA_* is the weisht fraction of PLLA content, *ΔH_m_^0^* is melting enthalpy of 100% crystalline PLLA (93.6 J g^−1^).^[^ ^40^ ^]^ Summary of thermal transition parameters determined by DSC analysis are provided in Table S1 (Supporting Information). The cold crystallization temperature (*T*_cc_) significantly decreases from 81.2 °C to 72.5–74.8 °C with the addition of PEG, indicating that PEG is compatible with PLLA. Additionally, the α‐phase crystallinity rate of PLLA content, analyzed through heat flow, was determined as 16.5%, 28.3%, 41.0%, and 44.4% for pure PLLA, PEG10, PEG20, and PEG30, respectively (Figure 2F). The formation of α‐phase crystalline PLLA is further evidenced by the XRD patterns shown in Figure S8 (Supporting Information), where an increase in peak intensity and a shift to a larger 2θ value indicate a higher α‐phase content and enhanced crystallinity, transitioning from the loosely packed α′‐phase to the denser α‐phase. Considering the PLLA fraction in the blend, PEG20 contains the highest α‐phase content among the specimens. Therefore, based on the mechanical properties, including a low Young's modulus with high elongation, and the α‐phase crystallinity achieved, PEG20 was selected as the optimal blend for developing our APNF‐NGC.

Cell adhesion, the initial stage of cellular growth, is mediated by the binding of integrins, and is generally enhanced on hydrophilic and negatively charged surfaces. However, the presence of non‐polar methyl groups in PLLA results in a hydrophobic surface on PLLA nanofibers. In our study, the incorporation of PEG's polar ether groups modifies the surface properties, influencing both the surface potential and hydrophilicity. To evaluate the effect of PEG blending, Kelvin probe force microscopy (KPFM) was utilized to examine the surface potentials of PLLA and PEG20 nanofiber strands. As shown in Figure 2H, the surface potentials of PLLA and PEG20 nanofiber strands were estimated at 553.1 and ‐49.89 mV, respectively, indicating a decrease of 603 mV due to PEG blending. Furthermore, hydrophilicity was assessed through contact angle measurements. PLLA and PEG20 nanofiber mats exhibited contact angles of 92.4 ° and 27.6 °, respectively (Figure 2I). Water absorption tests also confirmed the enhanced hydrophilicity of the PEG20 nanofiber mat, which showed a six‐fold increase in absorption compared to PLLA mat. (Figure S9, Supporting Information). Consequently, in terms of surface potential and hydrophilicity, PEG20 demonstrates more favorable surface properties conducive to cell adhesion. Furthermore, MTT assays were conducted to assess the biocompatibility of PLLA and PEG20 electrospun mats. Fibroblast cells were cultured for 3 days with and without the mats, and cell viability was evaluated relative to a control group in which cells were cultured in wells without any electrospun material (Figure S10, Supporting Information). For PLLA, the normalized MTT absorbance values were 105.3 ± 9.96%, 107.4 ± 4.74%, and 100.7 ± 6.73% on Day 1, Day 2, and Day 3, respectively. PEG20 showed similarly high values of 117.6 ± 16.18%, 111.3 ± 10.38%, and 103.9 ± 9.10% at the corresponding time points. No statistically significant differences were observed between any of the experimental groups and the control, as confirmed by unpaired Student's t‐tests (Figure S10, Supporting Information). These results collectively demonstrate that both PLLA and PEG20 provide a cytocompatible environment for cell proliferation, with no indication of material‐induced cytotoxicity.

**Figure 3:**
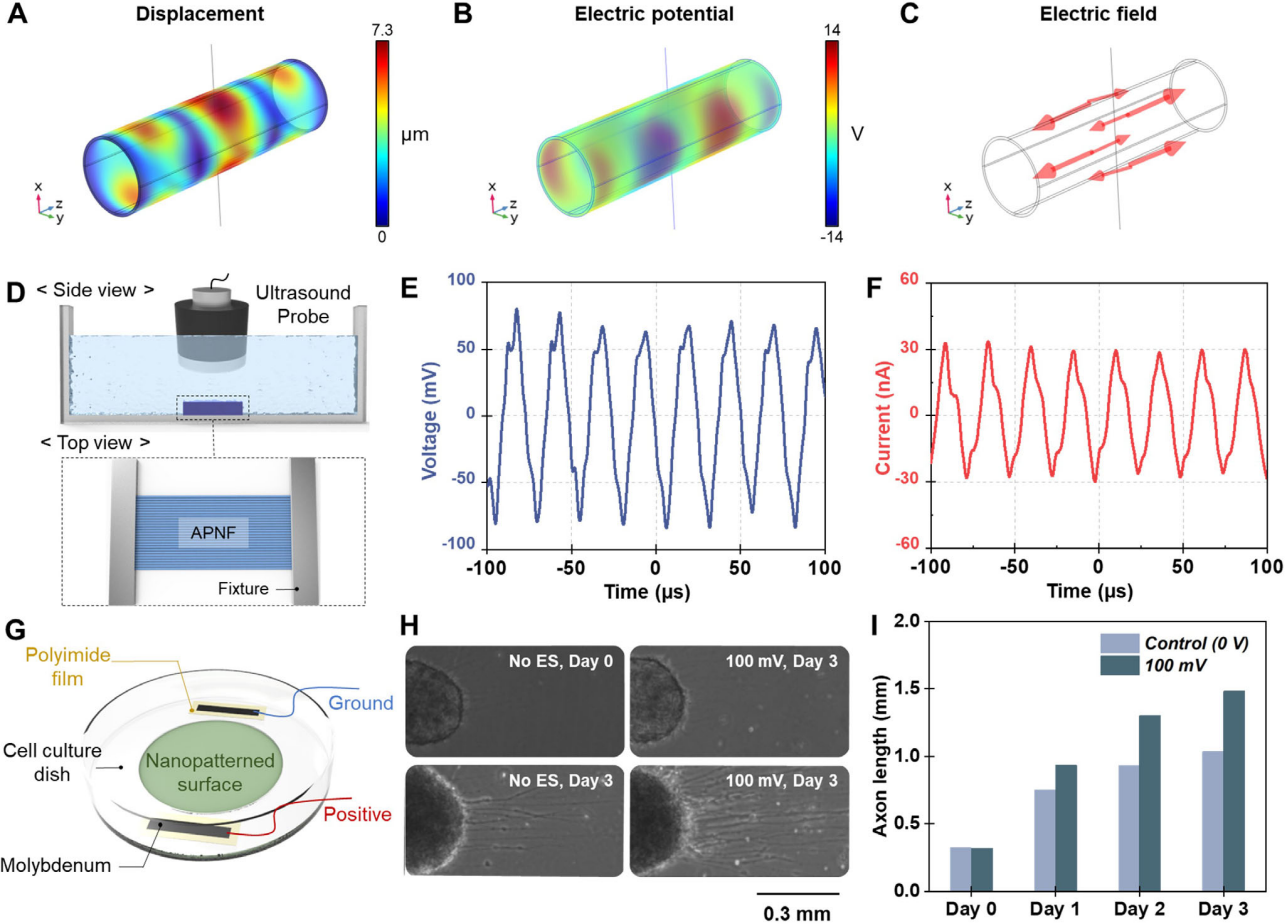
Piezoelectricity of APNF‐NGC. FEA of ultrasound‐induced dynamics of APNF‐NGC: A) displacement, B) piezoelectric potential, and C) electric field distribution. D) Schematic illustration of the electrical measurement setup for APNF under ultrasound. E) Voltage and F) current output of APNF under 40 kHz ultrasound. G) Schematic illustration of the in vitro experimental setup using iPSC‐derived motor neurons to validate the effect of electrical stimulation on nerve regeneration. H) Optical microscopy images showing axon elongation of iPSC‐derived motor neurons over 3 days with and without electrical stimulation (40 kHz, 100 mV AC; No ES = no electrical stimulation). I) Quantification of axon length measured over 3 days, indicating enhanced elongation under electrical stimulation.

In our design of the piezoelectric NGC, we aimed to utilize ultrasound‐induced piezoelectric response to electrically promote nerve regeneration. Ultrasound serves as an effective non‐invasive method for activating the implanted NGC due to its safety and deep tissue penetration. Under ultrasound, our APNF‐NGC is designed to generate an electric field in the axial direction to electrically guide the direction of axon elongation and Schwann cell growth. Our PLLA is in α‐phase crystalline, which has shear piezoelectricity from its helical arrangement of dipoles, and owing to the electrospinning method, the orientation of the molecular chain aligns with the nanofiber length direction. To confirm the ultrasound‐driven piezoelectricity, we performed an FEA‐based preliminary investigation of the acoustic dynamics of APNF‐NGC under irradiation of 40 kHz ultrasound. We have chosen 40 kHz which is a low frequency level facilitating large displacement of APNF for efficient piezoelectric generation. As shown in **Figure** **3**A, the ultrasound induces microscale displacements within the APNF‐NGC, with multiple axial nodes vibrating up and down due to the non‐uniform distribution of acoustic pressure and stress across the conduit (Figure S11, Supporting Information). These dynamics of APNF‐NGC yield alternating piezoelectric potential along the conduit, with the resulting electric field oriented in the axial direction (Figure 3B,C).

Subsequently, the electrical output of APNF was evaluated when subjected to ultrasound irradiation. The APNF mat was suspended with a metallic fixture in water to simulate implantation in the human body. Water served as an acoustic medium analogous to human tissue. The fixtures were positioned 8 mm apart to correspond with the nerve deficit length and arranged on two sides of the axial orientation of APNF (Figure 3D). Under 40 kHz ultrasound irradiation, the APNF mat generated a sinusoidal waveform with a peak‐to‐peak voltage (*V*_pp_) of 161.2 mV and a peak‐to‐peak current (*I*_pp_) of 58.7 nA, corresponding to root mean square (RMS) values of 46.7 mV and 18.4 nA, respectively (Figure 3E,F; Figure S12, Supporting Information). To validate the effect of APNF‐induced electrical stimulation on nerve regeneration, we conducted in vitro experiments using induced pluripotent stem cells (iPSC)‐derived motor neuron to assess the impact of in‐plane electric stimulation on nerve growth. The iPSC‐motor neurons were cultured on a cell dish, and subjected to sinusoidal electrical stimulation (40 kHz, 100 mV AC, 10 min per day) with observations recorded at 0, 24, 48, and 72 h (Figure 3G). The application of a 100 mV AC voltage resulted in significantly longer axon lengths compared to the control group without electrical stimulation (Figure 3H,I). These results demonstrate that ultrasound‐driven APNF‐NGC can provide sufficient electrical stimulation to promote nerve regeneration, particularly through axon elongation. This regenerative effect is attributed to acoustically actuated piezoelectric scaffolds generating localized electric fields that modulate neuronal and Schwann cell activities. In neurons, such electrical stimulation induces calcium influx through voltage‐gated Ca^2+^ channels, activating downstream signaling cascades including the calmodulin–adenylate cyclase–cyclic AMP–protein kinase A (cAMP/PKA) pathway, which upregulates growth‐associated proteins such as GAP‐43, critical for axonal elongation and guidance (Figure S13, Supporting Information).^[^ ^41^, ^42^ ^]^ Concurrently, Schwann cells respond to electrical stimulation via Ca^2+^‐mediated activation of the cAMP/PKA signaling pathway, enhancing their proliferation, migration, and myelination capacity. Furthermore, electrical cues stimulate the expression of neurotrophic factors such as nerve growth factor (NGF) and brain‐derived neurotrophic factor (BDNF), both of which contribute to axonal regeneration and remyelination processes.^[^ ^43^ ^]^ These cellular responses collectively support the therapeutic efficacy of ultrasound‐activated piezoelectric scaffolds in peripheral nerve repair.

**Figure 4:**
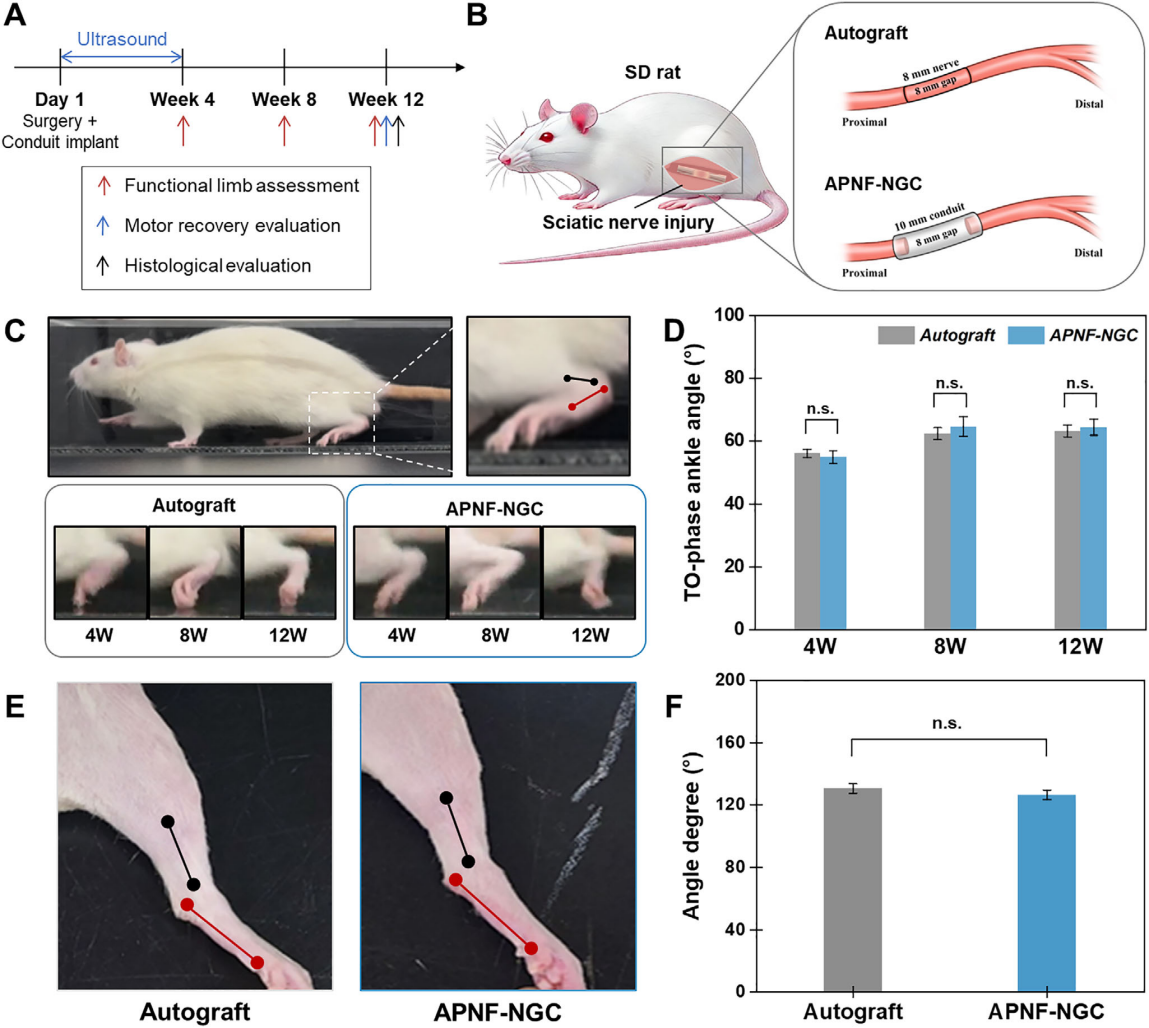
In vivo experiment of peripheral nerve repair using APNF‐NGC and evaluation of limb function recovery in sciatic nerve injury animal model. A) Schematic of the in vivo experimental timeline for APNF‐NGC. B) experimental group using SD rats with both 8mm‐sciatic nerve deficits: Autograft and NGC group. C) Video‐gait analysis for evaluation of the ankle angle at the toe‐off (TO) phase during gait. D) The TO phase angles measured every 4 weeks. E, F) Contraction angle measurement on 12 W by physically pulling the leg on the side. All the red marker dots (at the calcaneus, and fifth metatarsal head) and the black dots (at the proximal points of the lower third of the tibia and lateral malleolus) were used to measure the ankle and contracture angle between each point. The mean ± SEM (standard error of the mean) values of the Autograft and APNF‐NGC groups are compared (n.s. = not significant, *p* > 0.05; n = 15; Student's t‐test).

To investigate the therapeutic potential of the APNF‐NGC, we conducted in vivo experiments using a rat model with an 8 mm sciatic nerve defect. **Figure** **4**A outlines the experimental design and timeline. Two experimental groups were prepared for the study: an Autograft group and an APNF‐NGC group, each comprising 15 rats (Figure 4B; Figure S14, Supporting Information). The Autograft group served as the control to calibrate the efficacy of the APNF‐NGC, as autografts are the clinical “gold standard” for peripheral nerve repair. For the APNF‐NGC to be considered effective, its regenerative outcomes must be comparable to those of the autograft. These evaluations aim to determine whether APNF‐NGC can provide a viable alternative to autografts for peripheral nerve regeneration. In the Autograft group, the severed sciatic nerve was reversed and reimplanted to bridge the gap. Whereas, in the APNF‐NGC group, a 10 mm APNF‐NGC was implanted to connect the nerve stumps. After a 3‐day post‐surgery recovery period, the APNF‐NGC group received ultrasound stimulation at 40 kHz to induce the piezoelectric effect. The stimulation was applied for 30 min, three times per week, for four weeks (Figure S15, Supporting Information). Then, limb function, motor recovery, and histological assessments of both muscle and regenerated sciatic nerve tissue were conducted at specific time points to evaluate the extent of nerve regeneration. Throughout the study, the rats were housed in a standard environment, allowing free movement and access to regular food to maintain their physiological health.

To assess limb recovery during the gait cycle, we measured the ankle angle during the toe‐off (TO) phase at 4‐, 8‐, and 12 weeks post‐surgery. The ankle angle was defined as the intersection of lines extending from the knee to the ankle joint and from the ankle joint to the metatarsal head (Figure 4C; Figure S16, Supporting Information). The ankle angle fluctuates throughout the gait cycle, reaching maximum plantar flexion in the TO phase when the foot leaves the ground during normal gait.^[^ ^44^ ^]^ Sciatic nerve injury causes a reduction in TO‐phase ankle angles compared to normal gait, making this measurement a crucial marker for assessing gait disorders following peripheral nerve injury and repair.^[^ ^45^, ^46^, ^47^ ^]^ At 4, 8, and 12 weeks, the ankle angles during the TO phase were 56.2 ± 1.3 °, 62.4 ± 1.9 °, and 63.2 ± 1.9 °, respectively, in the autograft group, and 54.9 ± 2.1 °, 64.6 ± 3.1 °, and 64.4 ± 2.6 °, respectively, in the APNF‐NGC group (Figure 4D). Statistical analysis revealed no significant difference in gait‐related limb function between the two groups.

We also assessed limb function recovery by measuring the contracture angle of the operated leg. Sciatic nerve injuries often result in foot eversion and inversion deformities, caused by interphalangeal joint contractures and abnormal muscle reinnervation.^[^ ^48^ ^]^ Given the structural complexity of the sciatic nerve and the distance to its end‐organ targets, incomplete or misdirected reinnervation is common.^[^ ^49^ ^]^ As a result, contractures are frequent in sciatic nerve injury models due to residual muscle paralysis and imbalance.^[^ ^50^ ^]^ As shown in Figure 4E, the contracture angle was assessed by physically extending the operated leg under anesthesia. The contracture angle was defined as the intersection of two lines: one from the calcaneus to the fifth metatarsal head (red line) and the other between the lower third of the tibia and the lateral malleolus (black line). At 12 weeks, the contracture angles for the Autograft and APNF‐NGC groups were 130.5 ± 3.1 ° and 126.6 ± 3.0 °, respectively, with no statistically significant difference between the two groups (Figure 4F). Based on the results for both the TO phase‐ankle angle and the contracture angle, we conclude that there was no significant difference in limb function recovery between the two experimental groups. This suggests that the APNF‐NGC is as effective as the autograft in promoting limb function recovery.

**Figure 5:**
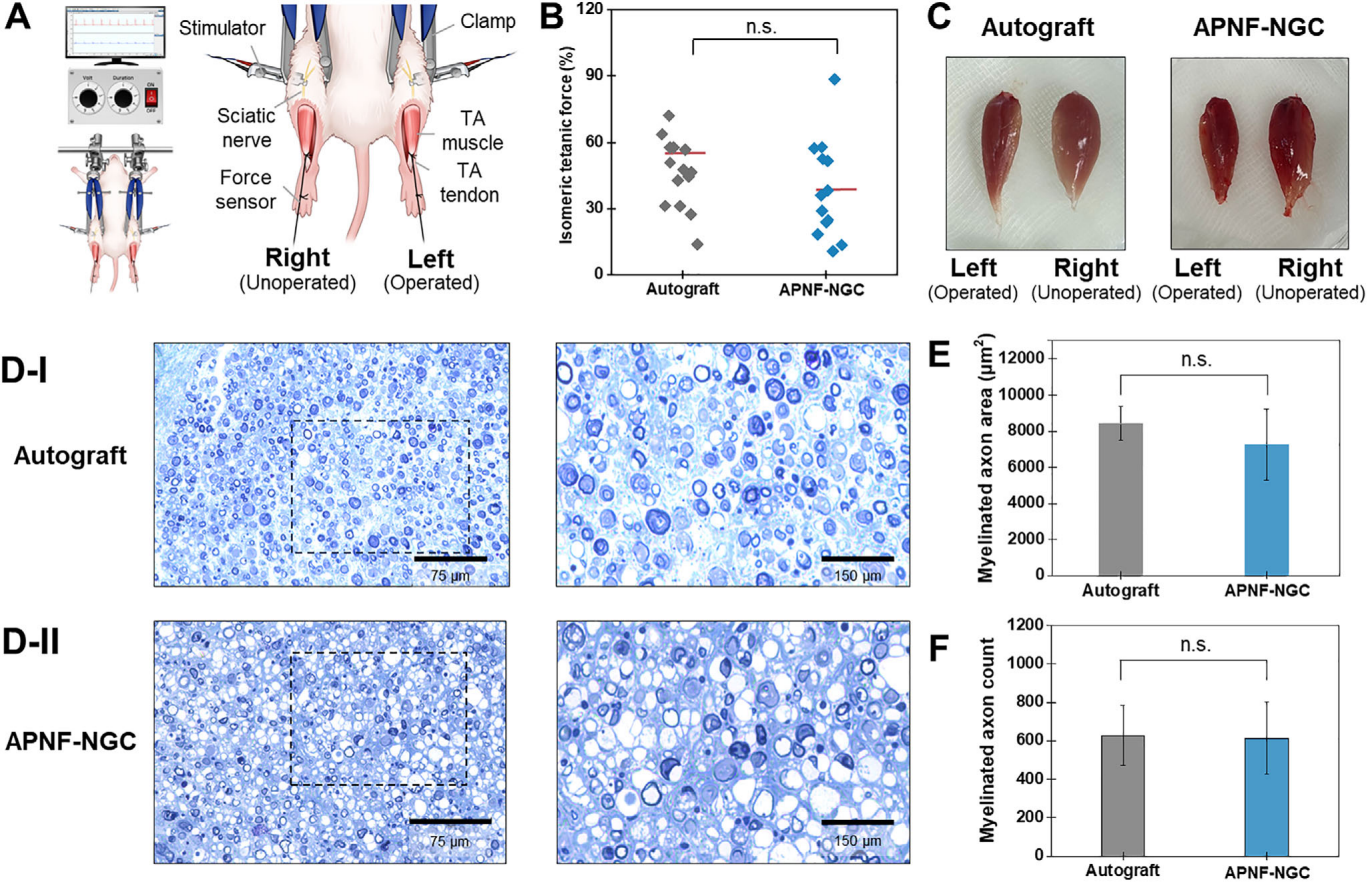
Electrophysical and histological evaluation of sciatic nerve injury model. A) Schematic view of the measurement for isometric tetanic force. B) Measurement of the isometric tetanic force were assessed at 12 weeks postoperatively. C) Photographs of isolated TA muscles measured at Week 12. TA muscle image of received surgery (Left) and normal (Right) side were D) Semi‐thin sections of the sciatic nerve toluidine blue staining for I) Autograft and II) APNF‐NGC groups. E) Myelinated axon area and F) Myelinated axon count calculated from semi‐thin sections. The mean ± SEM (standard error of the mean) values of the Autograft and APNF‐NGC groups are compared (n.s. = not significant, *p* > 0.05; n = 15; Student's t‐test).

Since the sciatic nerve branches into the tibial, sural, and common peroneal nerves, denervation leads to muscle atrophy and a loss of corresponding muscle weight. Consequently, assessing the tibialis anterior (TA) muscle serves as an important indicator of motor recovery following sciatic nerve injury.^[^ ^51^ ^]^ To evaluate the motor recovery of TA muscle function, we conducted an isometric tetanic force analysis at 12 weeks postoperatively.^[^ ^52^ ^]^ Under electrical stimulation applied to both the operated (left) and unoperated (right) sciatic nerves, the TA muscular contraction force was measured from the distal TA tendon while immobilizing the knee and ankle joints with clamps (**Figure** **5**A; Figure S17, Supporting Information). The ratio of the isometric tetanic force of the operated to the unoperated TA muscles was plotted as shown in Figure 5B. The Autograft and APNF‐NGC groups exhibited forces of 55.3 ± 15.7% and 38.6 ± 21.3%, respectively. Although the TA muscle contraction force in the APNF‐NGC group was lower than that of the Autograft group, no significant differences were observed between the groups based on p‐value analysis.

Subsequently, to visualize the TA muscle at 12 weeks, the muscle was collected after sacrifice, and the weight of the TA muscle was measured in individual animals to further evaluate motor recovery. The weight ratio of the left (operated) to the right (unoperated) TA muscles for the Autograft and APNF‐NGC groups was found to be 57 ± 3.6% and 43 ± 2.7%, respectively (mean ± SEM, n = 15, Figure 5C; Figure S18, Supporting Information)). Overall, while the APNF‐NGC does not restore TA muscle function to the same extent as autografts, previous studies have shown that conduits typically provide 30–50% less muscle recovery than autografts.^[^ ^53^, ^54^, ^55^ ^]^ Therefore, our APNF‐NGC demonstrates outstanding performance in motor recovery during nerve repair, as evidenced by the statistical interpretation of the TA muscle evaluation results, which showed no significant difference compared to the control group (Autograft).

Lastly, we conducted a histological evaluation of axon regeneration using toluidine blue (TB) staining at 12 weeks postoperatively. In our established animal model of sciatic nerve injury, transverse sections were obtained from the distal stump, ≈2–3 mm away, and fixed for analysis (Figure S19, Supporting Information). TB staining was employed to directly observe sciatic nerve axon regeneration in each group, and the slides were analyzed to quantify the myelinated axonal area, and total axon count (Figure 5D). The total area of the myelinated axon was 8425 ± 252 µm2 for the Autograft group and 7245 ± 527 µm^2^ for the APNF‐NGC group (Figure 5E). The number of myelinated axons was counted as 627.8 ± 41.8 in the Autograft group and 615 ± 49.9 in the APNF‐NGC group (Figure 5F). The results indicated that the APNF‐NGC group demonstrated comparable axon regeneration to the Autograft group, with no statistically significant difference in the myelinated axonal area or total axon count. Considering previous studies that have shown axon regeneration of NGCs typically exhibits a 40–50% lower extent compared to autografts, this finding suggests that the APNF‐NGC could serve as a promising alternative to autografts for peripheral nerve repair.^[^ ^56^, ^57^, ^58^ ^]^

## 3 Conclusion

In this study, we developed PLLA/PEG blend‐based APNF‐NGC featuring highly aligned morphology and wireless electrical stimulation for peripheral nerve repair. The electrospun APNF mat provides anisotropically oriented guidance and modulates Young's modulus and mechanical strength for a flexible and robust NGC. Furthermore, the incorporation of PEG enhanced the piezoelectric α‐phase PLLA content and modified the surface conducive to cell growth. 40 kHz ultrasound applied to the APNF mat confirms the wireless generation of an axial electric field in the axial direction of NGC sufficient to promote neuroregeneration. We further evaluated the efficacy of APNF‐NGC in a sciatic nerve injury model, utilizing autografts as a control group. Comprehensive assessments of limb function, muscle motor recovery, and neural tissue regeneration demonstrated that the APNF‐NGC achieved comparable regenerative outcomes to autografts, indicating its therapeutic viability as a substitute for traditional nerve grafts. Our APNF‐NGC is easily processible, adaptable in size and shape, and combines multiple regenerative mechanisms, highlighting its potential for broader applications in not only in large‐gap injury repair but also other types of tissue regeneration where electrical stimulation could play a supportive role, such as muscle and cardiovascular tissues. This study provides insights into the design of advanced scaffold materials tailored for nerve regeneration.

## 4 Experimental Section

### Electrospinning and Fabrication of APNF‐NGC Conduit

PLLA (Ingeo 4032D, NatureWorks, Mw = 200 kDa) and PEG (Sigma‐Aldrich, Mw = 400 Da) were commercially obtained. Dichloromethane (DCM) and dimethylformamide (DMF) were also purchased from Sigma‐Aldrich. For prepeparation of electrospinning solution, DCM and DMF were mixed in a 1:1 volume ratio and vortexed thoroughly. PLLA and PEG were then added to achieve a total polymer concentration of 14 wt% at weight ratios of 100:0, 90:10, 80:20, 70:30, and 60:40, denoted as PLLA, PEG10, PEG20, PEG30, and PEG40, respectively. The mixtures were dissolved using a magnetic stirrer at 120 °C for 6 h. The homogeneous PLLA/PEG solution was loaded into a 10‐mL syringe equipped with a 21‐gauge stainless steel needle. Electrospinning was performed using a customized machine (ESR200RD, Nano NC) at room temperature with a relative humidity of ∼40%. The solution was injected at a flow rate of 30 µL min^−1^ using a syringe pump. A high voltage of 18 kV DC was applied to the needle tip, positioned 18 cm away from a grounded drum collector. The drum collector (diameter: 18 cm) was rotated at speeds of 100, 500, 1000, 1500, and 2000 rpm to control nanofiber orientation.

To fabricate the four‐channel APNF‐NGC, PEG20 electrospun mats were first cut into small pieces (6 mm × 8 mm × 35 µm). Each piece was tightly rolled around a 0.46 mm‐diameter stainless steel rod for two turns and annealed at 100 °C to fix the cylindrical shape, forming individual small channels. Four of these small tubular structures were then aligned in parallel. An additional PEG20 mat (12 mm × 10 mm × 125 µm) was used to wrap around the bundled four channels, and the entire assembly was annealed again at 100 °C to retain the final conduit shape. The thermal stability of the PEG20 mat was confirmed by thermogravimetric analysis (Figure S20, Supporting Information). All conduits were sterilized using ethylene oxide (EO) gas sterilization under standard conditions before implantation.

### Material Characterization

X‐ray diffraction (XRD) analysis was performed using a SmartLab diffractometer (Rigaku, Japan) equipped with Cu Kα radiation (λ = 1.5418 Å), operated at 40 kV and 30 mA. The diffraction patterns were collected in the 2θ range of 5–40°, with a step size of 0.02° and a scan rate of 2° min^−1^. Differential scanning calorimetry (DSC) was conducted using an SDT Q600 thermal analyzer under a nitrogen atmosphere. Samples of 5–10 mg were heated from 20 to 200 °C at a rate of 10 °C min^−1^. Surface potential measurements were conducted with a Kelvin probe force microscopy (KPFM) module integrated into an atomic force microscope (AFM, XE‐100, Park Systems), using a Pt/Cr‐coated silicon tip. Contact angle measurements were performed using 100 µL DI water droplets on nanofiber mats, and the angle was quantified using ImageJ software. The mechanical properties of the electrospun mats were evaluated using a universal testing machine (AGX‐20kNVD, Shimadzu) equipped with a 50 N load cell. Mechanical testing was conducted at a strain rate of 5 mm min^−1^ at room temperature, using electrospun mat specimens with a dimension of 1 cm × 4 cm × 50 µm.

### Electrical Characterization

The voltage output was measured using an oscilloscope (Tektronix, DPO3052) and a 40 MΩ input impedance probe (Tektronix, P5100A). The current output was measured using a low‐noise current amplifier (FEMTO, DLPCA‐200) connected to the oscilloscope. A 40 kHz ultrasonic transducer (LW‐101) was used to apply acoustic stimulation at an intensity of 0.75 W cm^−2^. The probe frame was grounded to reduce electrical noise. The APNF mat was placed 5 mm from the ultrasound probe, and its ends were fixed by metal fixtures to allow free‐standing vibration and ensure proper electrical contact.

### Finite Element Analysis (FEA)

Numerical simulations were performed using COMSOL Multiphysicsto evaluate the acousto‐piezoelectric behavior of the APNF‐NGC under ultrasound stimulation. A multiphysics model coupling pressure acoustics, solid mechanics, and electrostatics was employed. The simulation geometry consisted of a cylindrical conduit (outer diameter: 1.5 mm; wall thickness: 0.2 mm; length: 10 mm) embedded within a cubic water domain (50 × 50 × 50 mm^3^). A circular ultrasound transducer (30 mm diameter) was placed 5 mm above the conduit. An incident pressure wave with a peak amplitude of 500 kPa and a frequency of 40 kHz was applied at the transducer surface. Perfectly matched layers (PMLs) were assigned to the outer water boundaries to minimize wave reflection. Material properties for the conduit were assigned based on literature values, including the elastic modulus, Poisson's ratio, piezoelectric coefficients, and dielectric permittivity.^[^ ^18^ ^]^ A fine tetrahedral mesh with a minimum element size of 0.01 mm was used to ensure numerical convergence in resolving the displacement and electric potential distributions.

### Animal Model and Surgery Procedure

This study followed the Animal Research Reporting or In Vivo Experiments (ARRIVE) guidelines. The Institutional Animal Care and Use Committee of Asan Medical Center and Ulsan University College of Medicine approved all animal care and experimental procedures (IACUC‐2023‐30‐123), and all the procedures provided below were carried out under the applicable guidelines and regulations. A total of thirty adult male Sprague‐Dawley rats (7 weeks old, OrientBio, Gapyong, Gyeonggi‐do, Korea) were randomly divided into two groups at random: the Autograft group (n = 15) and the piezoelectric group (n = 15). After anesthetization by intramuscular injection of 20 mg kg^−1^ Zoletil (Virvac Laboratories, Carros, France), the left sciatic nerve of the rats was exposed. In the case of the Autograft group, the sciatic nerve was cut 8 mm in length and the sciatic nerve was transplanted flipped upside down. In the piezoelectric group, the sciatic nerve was cut 8 mm in length and a 10 mm piezoelectric nerve conduit was implanted to cover the sciatic nerve. All surgery was conducted under a microscope and the 9‐0 nylon (Ethicon, Somerville, NY) was used for nerve sutures. The skin incision was sutured with 4‐0 nylon (Ethicon, Somerville, NY). One week post‐surgery, only the piezoelectric group underwent anesthesia with isoflurane (2‐3% in O_2_) and received 40 kHz ultrasound treatment (LW‐101) three times per week for 30‐min sessions at an intensity of 0.75 W cm^−2^.

### Video Gait Ankle Angle Analysis

Video gait analysis is a method for analyzing rat joint kinematics with a video camera positioned perpendicular to the direction of rat movement (sagittal plane), with the assumption that rat hindlimb movements are planar. A walking track (length 1 m, width 10 cm, and height 10 cm) was built for this test. The video was recorded for the video gait analysis using a digital camera (Canon SX730HS, Canon, Tokyo, Japan) at 1 meter, the camera was calibrated to prevent any optical distortion and records were repeated until three satisfactory trials were obtained per rat. The ankle angle was measured at maximal plantar flexion during the TO phase in the experimental lateral ankle joint and walking was repeated until 3 or 4 satisfactory gaits per rat (without pause) were obtained. Then displayed in degrees once the foot and leg segments were manually identified in the video frames.

### Isometric Tetanic Force

To expose and transect the TA tendon distally, another skin incision was made anterior to the ankle. The knee and ankle joints were immobilized to a platform, and the TA tendon was connected to a force transducer using a customized clamp. A bipolar stimulator (Grass S88, Grass Instrument Corp, Quincy, MA) was used to generate stimulus and processed on a computer using LabVIEW software (National Instruments, Austin, TX). All contractions were performed at 4‐voltage to ensure maximal activation of all TA motor units and muscle strength was standardized as a percentage of the value obtained from the contralateral side.

### Analysis Using Toluidine Blue Staining

A distal sciatic nerve segment from the surgery site to 2–3 mm length was extracted from each group and fixed in 2.5% glutaraldehyde solution, then fixed in 1% osmium tetroxide, dehydrated in ethanol, and embedded in EPON resin (Miller‐Stephenson Chemical Co., Sylmar, CA, USA). To visualize myelin under light microscopy, cross‐sections 1 µm thick were stained with toluidine blue (Toluidine blue‐O; Daejung Chemicals & Metals, Seoul, Korea) Each section was first scanned at low magnification (×40), and representative fields were randomly selected using a coordinate generator in ImageJ software (National Institutes of Health, Bethesda, MD) to minimize selection bias. Digital images were then acquired at ×400 magnification from 4–6 non‐overlapping fields per sample. Myelinated axons were manually counted in each image. The total number of myelinated axons was manually counted in each image. Axonal density was calculated as the number of axons per mm^2^. For axonal area measurements, individual axons were manually traced using the freehand selection tool in ImageJ software, and the mean axonal area was computed for each group.

### Statistical Analysis

All experiments were performed with n = 15 rats per group, and measurements were repeated at least three times per sample to ensure reliability. The mean of the repeated measurements was calculated and used for statistical analysis. Animal IDs were randomly assigned and encoded by an independent researcher who was not involved in any data collection or analysis. These anonymized IDs were used throughout the experimental procedures and data analysis. Group allocation was only revealed after all data had been finalized. All data were presented as mean ±standard error of the mean (SEM). The differences and significance were verified using the Student's t‐test in GraphPad PRISM 9.0 software (GraphPad Software, La Jolla, CA, USA). The *P*‐value of <0.05 was statistically significant.

## Conflict of Interest

The authors declare no conflict of interest.

## Supporting information



Supporting Information

Supporting Information

## Data Availability

The data that support the findings of this study are available from the corresponding author upon reasonable request.
