# Supplementary material for: Wireless Acousto‐Piezoelectric Conduit with Aligned Nanofibers for Neural Regeneration
Source: Adv Mater. 2025 Sep 2;37(47):e03343. doi: 10.1002/adma.202503343 (PMC12651110; doi:10.1002/adma.202503343)
Supplement: Supplementary file 1 — Supporting Information [file ADMA-37-e03343-s002.docx]

Supporting Information

Wireless Acousto-Piezoelectric Conduit with Aligned Nanofibers for Neural Regeneration

Sera Jeon^†^, Dabin Kim^†^, Min-Young Jo^†^, Chae-Min Ryu, Daniel Sanghyun Cho, Byung-Ok Choi, Jae Kwang Kim*, Miso Kim*, and Sang-Woo Kim*

TABLE OF CONTENTS

**Supporting methods** FFT analysis of fiber alignment, MTT assay, Axon growth evaluation in iPSC-Derived Motor Neurons

**Figure S1.** Photograph of electrospinning setup to synthesize the APNF.

**Figure S2.** SEM images of PLLA/PEG electrospun products at various PLLA to PEG weight ratios of A) 100:0, B) 80:20, C) 60:40, D) 40:60, and E) 20:80 (scale bar = 10 µm).

**Figure S3.** SEM images of electrospun PLLA nanofibers depending on the rpm of drum collector. A) 100 rpm, B) 500 rpm, C) 1000 rpm, D) 1500 rpm, E) 2000 rpm. (Scale bar = 10 µm).

**Figure S4.** Fast Fourier transformation (FFT) analysis on SEM images of electrospun PLLA nanofibers depending on the different rpm of drum collector to assess the nanofiber alignment A) 100 rpm, B) 500 rpm, C) 1000 rpm, D) 1500 rpm, E) 2000 rpm.

**Figure S5.** Analysis on the distribution of fiber diameter. A) SEM image of PEG20 (scale bar = 20 µm). B) Histogram graph of fiber diameter distribution quantified from the SEM image.

**Figure S6.** X-ray diffraction (XRD) patterns of electrospun PLLA nanofibers in random and aligned arrangements corresponding to drum rotations at 100 and 2000 rpm, respectively.

**Figure S7.** Molecular structure displaying shear piezoelectricity (*d*_14_) of α-PLLA.

**Figure S8.** XRD patterns of PLLA/PEG electrospun mat with different PEG fraction.

**Figure S9.** Water absorption capacity of PLLA and PEG20 electrospun mat.

**Figure S10.** MTT assay results showing normalized MTT absorbance (%) of fibroblasts cultured on PLLA and PEG20 nanofiber mats over 3 days (24, 48, and 72 hours). Electrospun mats (3 × 3 mm^2^) were placed in 96-well plates, and absorbance was measured at 570 nm after formazan formation. Values were normalized to a control group cultured without any nanofiber samples and expressed as a percentage. The MTT assay reflects mitochondrial enzyme activity and is commonly used to assess cellular metabolic function. The mean ± SEM values of the Autograft and APNF-NGC groups are compared (values are mean ± SEM; n.s. = not significant, p > 0.05; *p < 0.05; n = 3; Student’s t-test).

**Figure S11.** Finite elemental analysis on APNF-NGC under 40 kHz ultrasound. A) Acoustic pressure acting on APNF-NGC. B) Stress distribution of APNF-NGC with as response to 40 kHz ultrasound.

**Figure S12.** Electrical evaluation of APNF devices to assess robustness and repeatability. (A) Voltage output of APNF device #1 under continuous ultrasound stimulation (40 kHz, 0.75 W cm^-2^), measured at 0, 20, and 40 minutes, demonstrating temporal stability. (B) Voltage output from APNF devices (#2–#5), showing consistent piezoelectric performance across samples. All measurements were conducted under identical ultrasound conditions.

**Figure S13.** Voltage-gated Ca^2+^ channel activation by piezoelectric stimulation.

**Figure S14.** Photographs illustrating the implantation of A) the autograft and B) the APNF-NGC in the injured sciatic nerve.

**Figure S15.** Photograph depicting the application of 40 kHz ultrasound to the APNF-NGC group during in vivo test. The SD rat is under anesthesia via inhalation of isoflurane (2-3% in O_2_), maintained with a nose cone.

**Figure S16.** Captured video images from gait analysis used to measure the ankle angle during the toe-off phase, assessing limb function recovery in A) Autograft and B) APNF-NGC groups. Videos were recorded every 4 weeks post-surgery to monitor functional recovery.

**Figure S17.** The photograph of the setup for measuring isometric tetanic force to evaluate the motor recovery.

**Figure S18.** Quantitative comparison of tibialis anterior (TA) muscle weight between Autograft and APNF-NGC groups (values are mean ± SEM; **p < 0.01; n = 15; Student’s t-test).

**Figure S19.** Schematic illustration for cutting & staining site for histologic analysis. We retrieved the transverse nerve segment from the 2-3 mm distal to the distal margin of the nerve graft.

**Figure S20.** Thermogravimetric analysis (TGA) of PLLA/PEG electrospun mat with different PEG fraction.

**Table S1.** Thermal behaviors and crystallinity of PLLA/PEG nanofibers determined by differential scanning calorimetry analysis.

**Supporting methods**

*FFT analysis of fiber alignment:* SEM images presented in Figure S3 were first obtained for each drum rotation speed (100, 500, 1000, 1500, and 2000 rpm). To enhance the clarity of the FFT results, the contrast of each SEM image was adjusted using ImageJ software prior to FFT transformation. After performing the FFT, the center of the FFT-transformed image was defined, and a fixed radius was set. Pixel intensities were then extracted along a circular path at every azimuthal angle (0–359°), and each intensity value was assigned to its corresponding angle. To minimize noise, the extracted data were smoothed using a moving average filter. Finally, the FFT intensity was plotted as a function of the azimuthal angle to quantitatively analyze the anisotropic features of the nanofiber alignment.

*MTT assay:* As part of the biocompatibility evaluation, mitochondrial activity of human fibroblast cells (ATCC CRL-1502) cultured on the scaffolds was assessed using an MTT assay. Fibroblasts were cultured in Dulbecco’s Modified Eagle Medium (DMEM) supplemented with 10% fetal bovine serum (FBS) and 1% penicillin–streptomycin. Electrospun mats were cut into 3×3 mm^2^ pieces and placed in each well of a 96-well plate. Fibroblasts (10^4^ cells per well) were seeded onto each sample and incubated for 24, 48, and 72 hours. After each time point, 50 µL of MTT solution (0.5 mg/mL, Thermo Fisher Scientific) was added and incubated for 2 hours at 37 °C under 5% CO_2_. The MTT solution and culture medium were then removed, and 150 µL of DMSO was added to dissolve the formazan crystals. Subsequently, 100 µL of the resulting solution was transferred to a fresh 96-well plate, and absorbance was measured at 570 nm using a microplate reader. A control group was included in which cells were seeded in wells without any electrospun sample. Absorbance values were normalized to this control group and expressed as normalized MTT absorbance (%).

*Axon growth evaluation in iPSC-Derived Motor Neurons:* This study, approved by the Institutional Review Board of Samsung Medical Center at Sungkyunkwan University (Approval No: 2021–04–053–001), involved the generation and differentiation of human induced pluripotent stem cells (iPSCs). Fibroblasts were isolated from skin tissue and cultured for approximately two weeks, followed by several subculturing steps to establish a uniform population.

Reprogramming fibroblasts was performed using the CytoTune-iPS 2.0 Sendai Reprogramming Kit (Invitrogen) along with defined transcription factors. The kit was utilized following the instructions from the manufacturer to induce the expression of four reprogramming factors: OCT3/4, KLF4, SOX2, and cMYC. After an additional two-week incubation, colonies with embryonic stem cell-like morphology were selected and the iPSCs were then expanded under feeder-free conditions using mTeSR medium (STEMCELL Technologies).

Before motor neuron differentiation, iPSCs in Essential 8 medium (Thermo Fisher Scientific, Waltham, MA, USA) in a sterilized vial were stored under cryogenic conditions in liquid nitrogen. For culture, cell vials were thawed in a 37 °C water bath for 3 min, and 1 mL of the cell suspension was transferred into a 15 mL conical tube. The volume was adjusted to 5 mL using Essential 8 medium containing 10 µM Y-27632 (Tocris Bioscience), a selective Rho-kinase inhibitor. After centrifugation at 300×*g* for 3 min, the supernatant was aspirated and the cells were resuspended in fresh medium with Y-27632. Cells were then seeded onto Matrigel-coated 6-well plates (Matrigel diluted 1:60 in DMEM/F-12, incubated at 37 °C/5% CO_2_ overnight). Y-27632 was removed 24 hours after plating, and cells were subsequently maintained in Essential 8 medium with daily medium changes.

Once the cells reached approximately 80% confluence, neural induction was initiated using a monolayer protocol to generate regionally unspecified neural progenitor cells.^[1]^ For passaging, the cells were seeded onto culture surfaces pre-treated with 0.01% poly-L-ornithine and 5 µg/mL laminin (Sigma-Aldrich). These cells were subsequently directed toward a motor neuron fate using a defined maturation protocol, typically involving extended culture in the presence of neurotrophic factors (BDNF, GDNF, CNTF) for an additional 2–3 weeks.^[2, 3]^ Using this differentiation method, approximately 30–40% of the resulting cell population expressed the standard motor neuron marker Islet-1. To improve purity, a negative selection fluorescence-activated cell sorting (FACS) procedure was performed on day 25 post-induction using antibodies against CD184 and CD44 (BD Biosciences, Franklin Lakes, NJ, USA) to eliminate non-neuronal cells.^[4]^ As a result, the proportion of Islet-1–positive cells increased to approximately 70%. Following FACS, neurons were replated at a density of 100,000 cells cm^-2^ and maintained in culture until day 40–50 post-induction, at which point they were used for terminal analyses. All cells used in the described experiments were differentiated from iPSC colonies between passages 45 and 55.

The resulting cells were seeded on nano-patterned culture dishes (Curibio Co. Ltd.) designed to support directional current flow. Electrodes were connected to both ends of the dishes, and electrical stimulation was applied using a function generator at a frequency of 40 kHz and amplitude of 100 mV for 10 minutes per day over three consecutive days. Following stimulation, axon length was assessed.

**
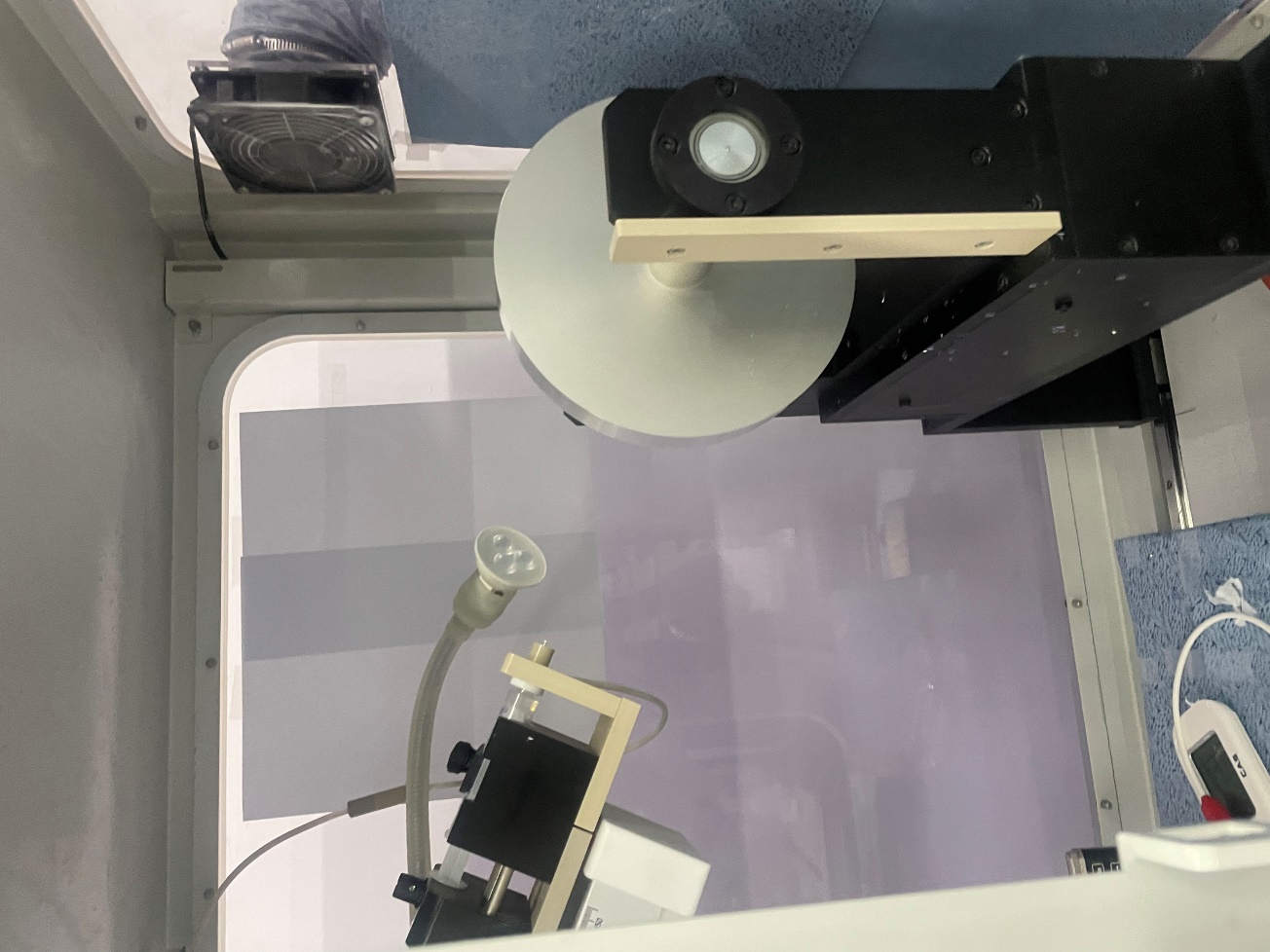
**

**Figure S1.** Photograph of electrospinning setup to synthesize the APNF.


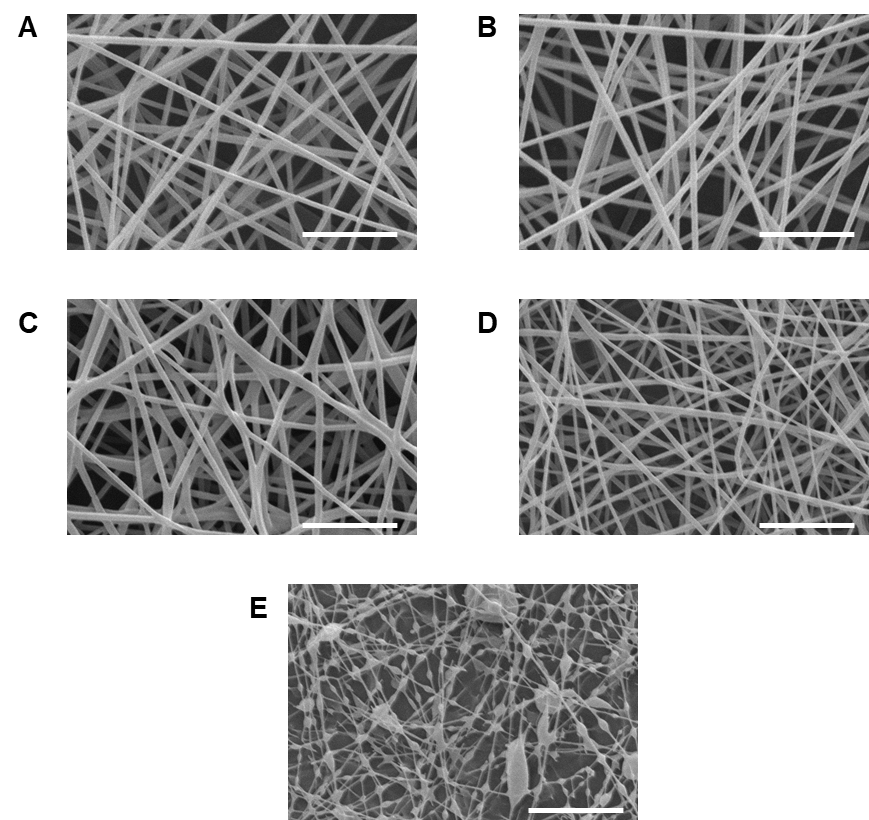


**Figure S2.** SEM images of PLLA/PEG electrospun products at various PLLA to PEG weight ratios of A) 100:0, B) 80:20, C) 60:40, D) 40:60, and E) 20:80 (scale bar = 10 µm).

**
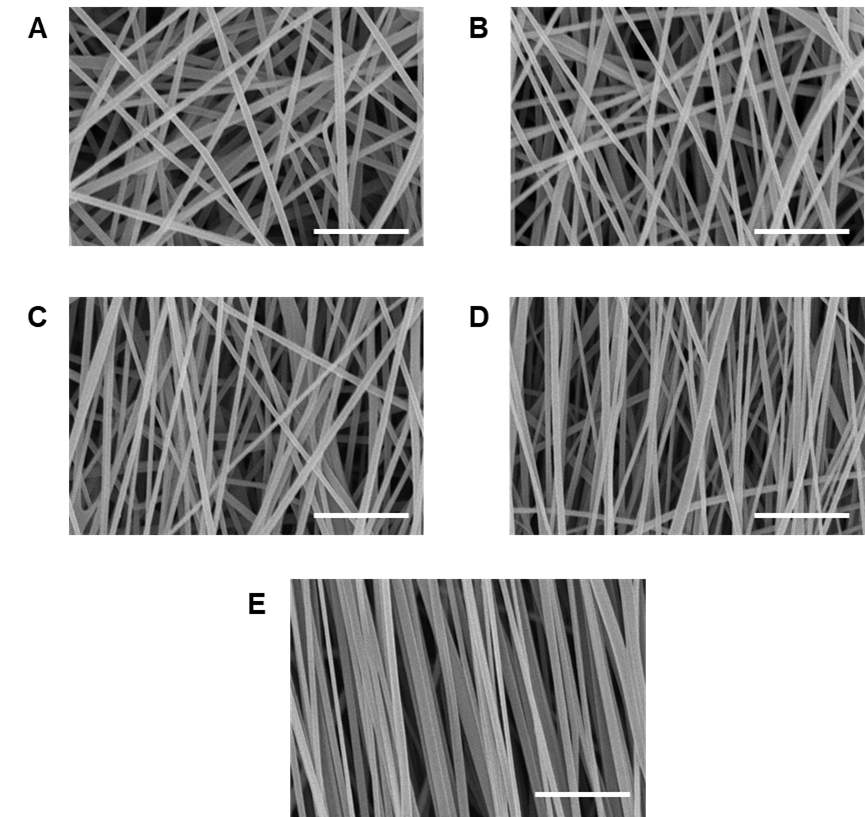
**

**Figure S3.** SEM images of electrospun PLLA nanofibers depending on the rpm of drum collector. A) 100 rpm, B) 500 rpm, C) 1000 rpm, D) 1500 rpm, E) 2000 rpm. (Scale bar = 10 µm).


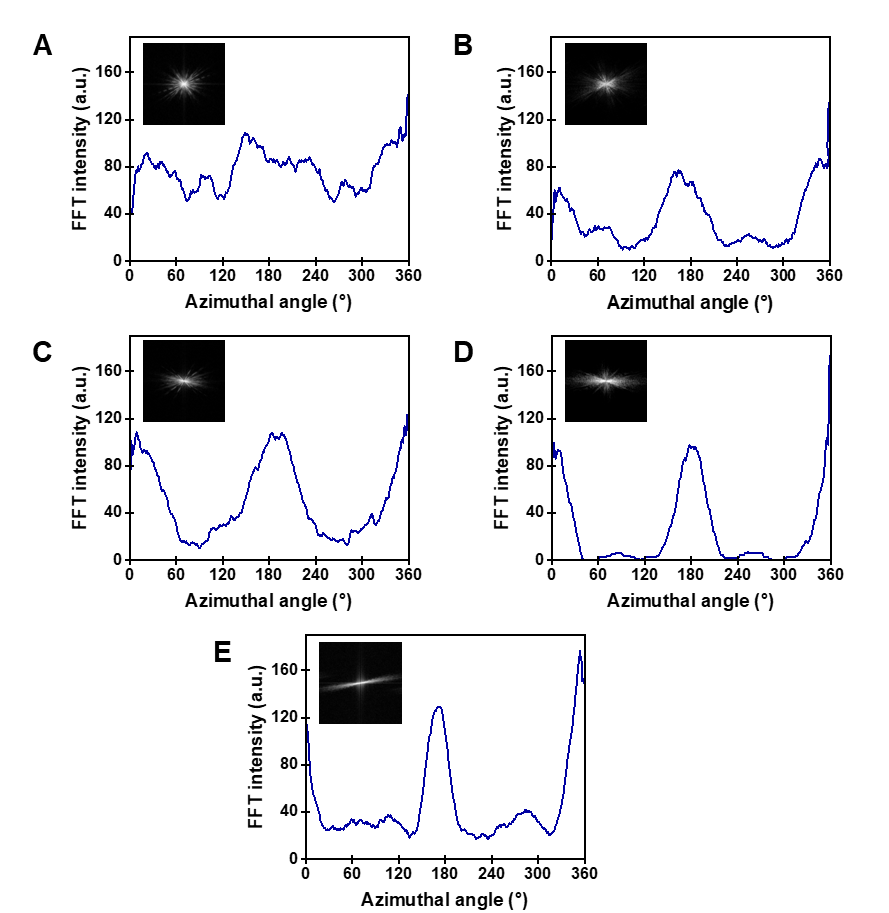


**Figure S4.** Fast Fourier transformation (FFT) analysis on SEM images of electrospun PLLA nanofibers depending on the different rpm of drum collector to assess the nanofiber alignment A) 100 rpm, B) 500 rpm, C) 1000 rpm, D) 1500 rpm, E) 2000 rpm.


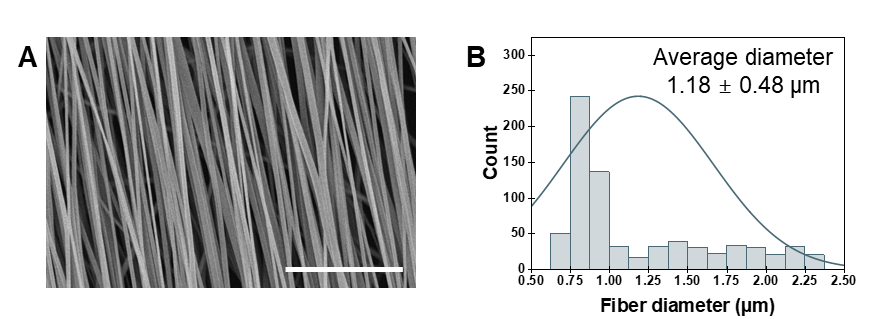


**Figure S5.** Analysis on the distribution of fiber diameter. A) SEM image of PEG20 (scale bar = 20 µm). B) Histogram graph of fiber diameter distribution quantified from the SEM image.

**Figure S6.** X-ray diffraction (XRD) patterns of electrospun PLLA nanofibers in random and aligned arrangements corresponding to drum rotations at 100 and 2000 rpm, respectively.


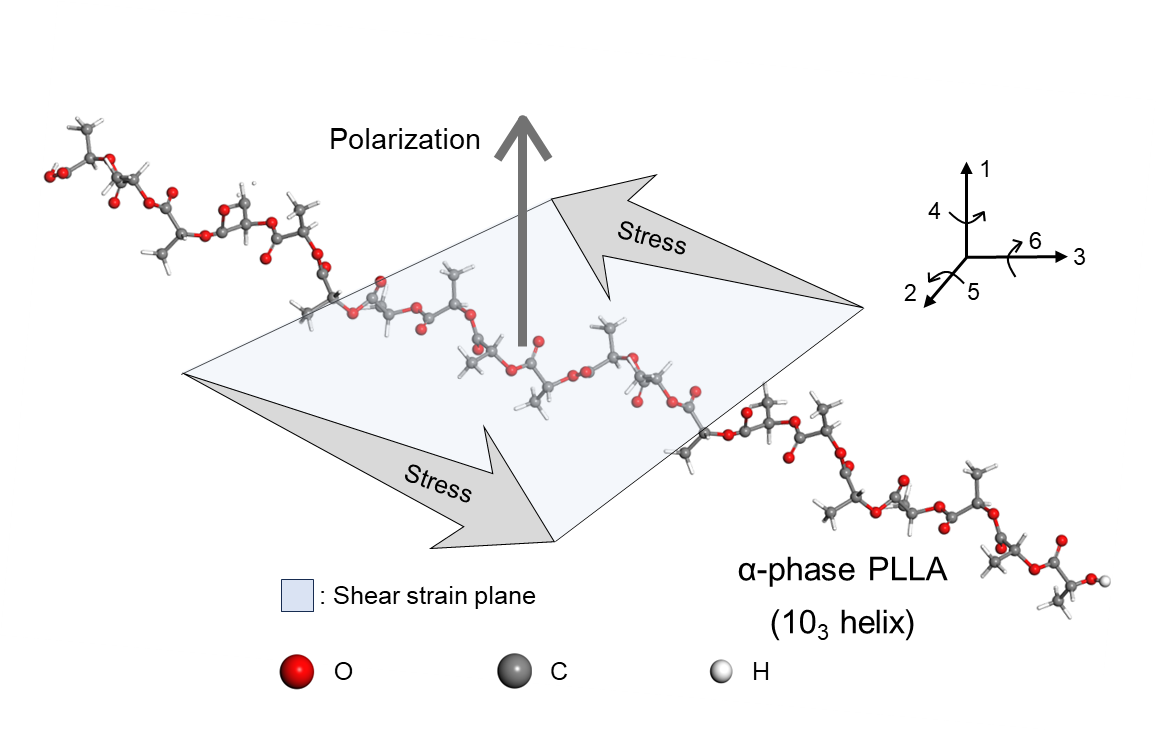


**Figure S7.** Molecular structure displaying shear piezoelectricity (*d*_14_) of α-PLLA.


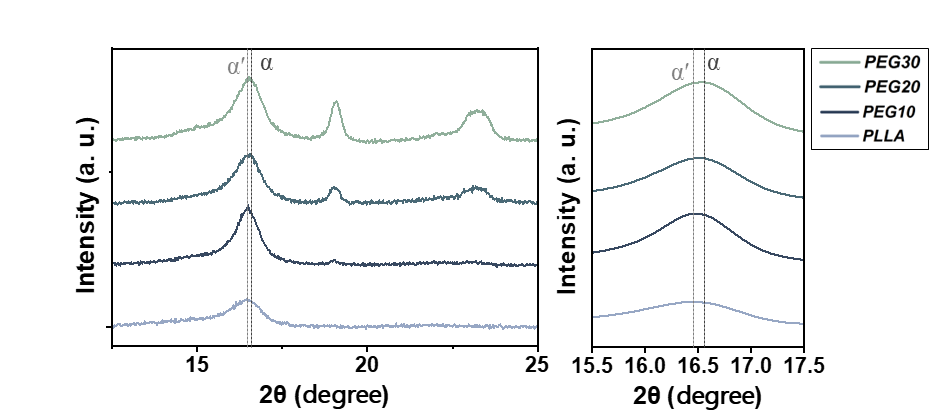


**Figure S8.** XRD patterns of PLLA/PEG electrospun mat with different PEG fraction.


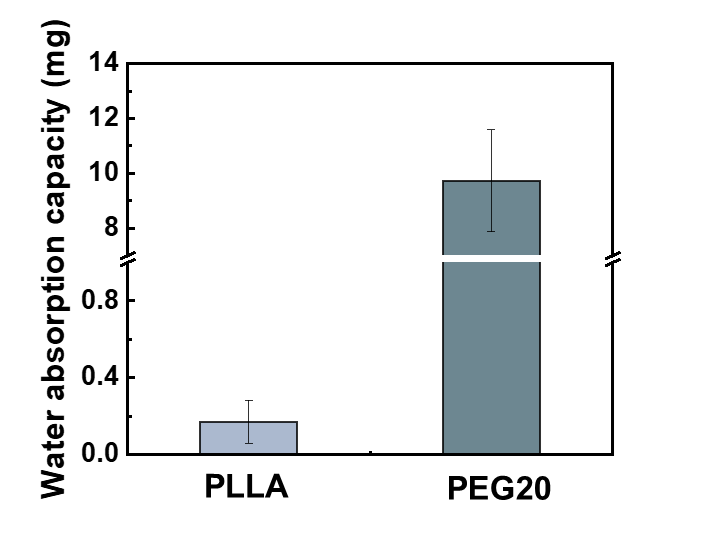


**Figure S9.** Water absorption capacity of PLLA and PEG20 electrospun mat.


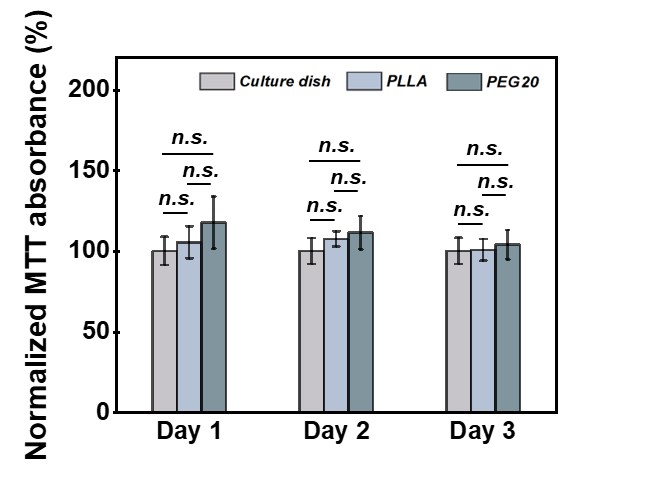


**Figure S10.** MTT assay results showing normalized MTT absorbance (%) of fibroblasts cultured on PLLA and PEG20 nanofiber mats over 3 days (24, 48, and 72 hours). Electrospun mats (3 × 3 mm^2^) were placed in 96-well plates, and absorbance was measured at 570 nm after formazan formation. Values were normalized to a control group cultured without any nanofiber samples and expressed as a percentage. The MTT assay reflects mitochondrial enzyme activity and is commonly used to assess cellular metabolic function. The mean ± SEM values of the Autograft and APNF-NGC groups are compared (values are mean ± SEM; n.s. = not significant, p > 0.05; *p < 0.05; n = 3; Student’s t-test).


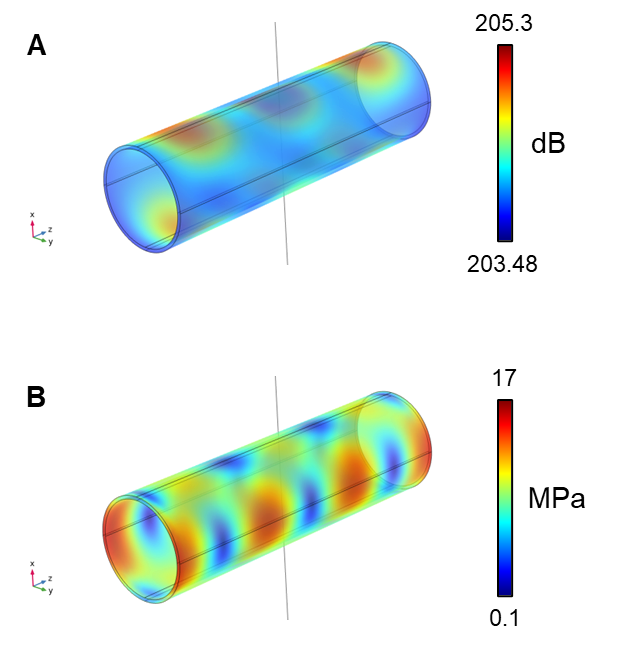


**Figure S11.** Finite elemental analysis on APNF-NGC under 40 kHz ultrasound. A) Acoustic pressure acting on APNF-NGC. B) Stress distribution of APNF-NGC with as response to 40 kHz ultrasound.

**
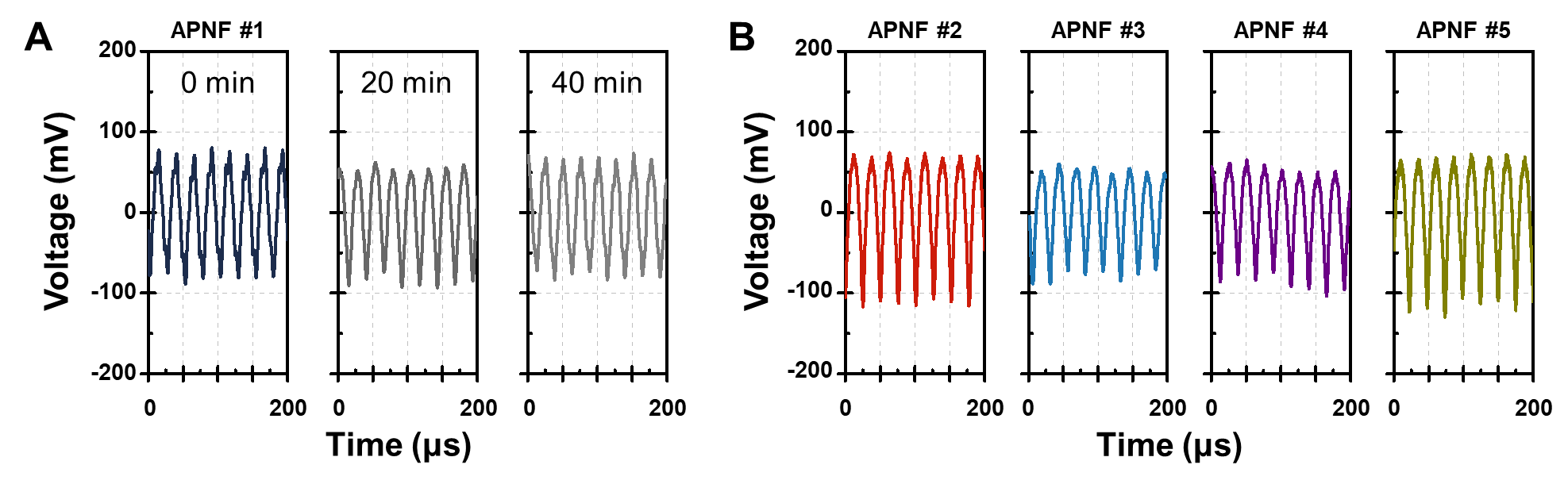
**

**Figure S12.** Electrical evaluation of APNF devices to assess robustness and repeatability. (A) Voltage output of APNF device #1 under continuous ultrasound stimulation (40 kHz, 0.75 W cm^-2^), measured at 0, 20, and 40 minutes, demonstrating temporal stability. (B) Voltage output from APNF devices (#2–#5), showing consistent piezoelectric performance across samples. All measurements were conducted under identical ultrasound conditions.


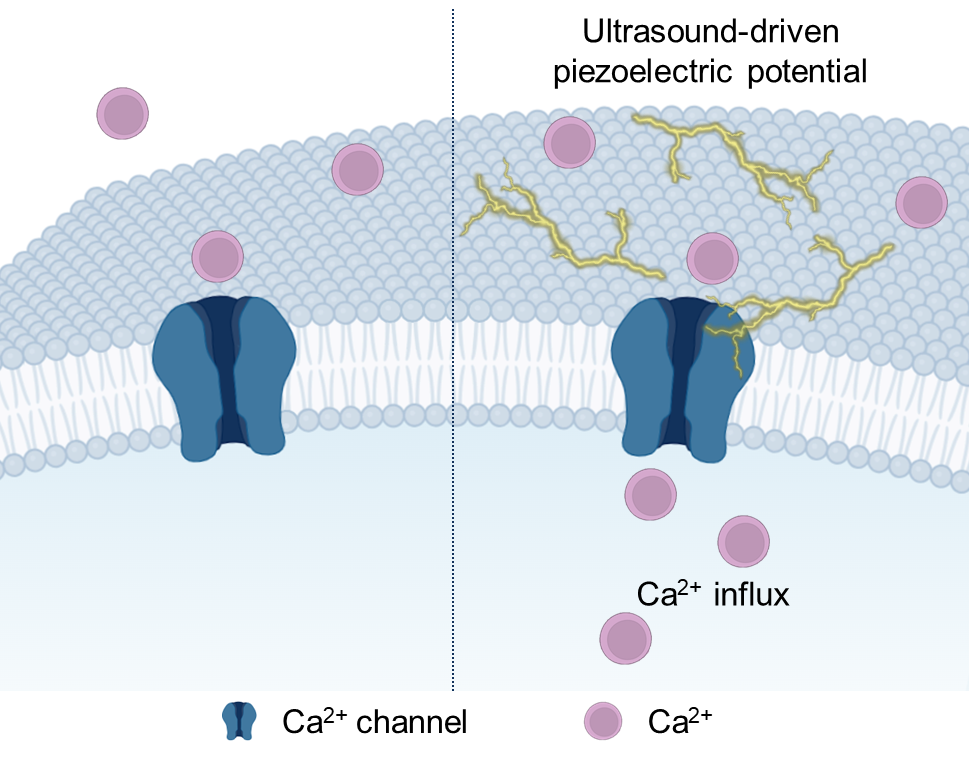


**Figure S13.** Voltage-gated Ca^2+^ channel activation by piezoelectric stimulation.


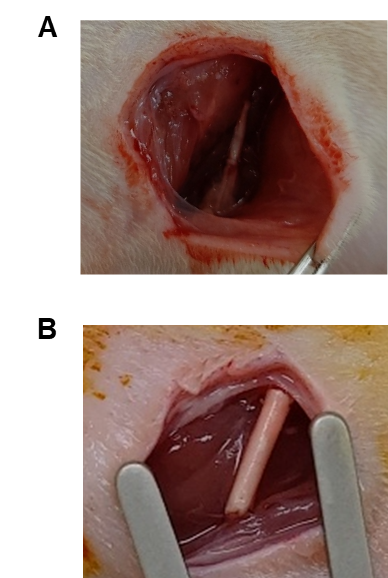


**Figure S14.** Photographs illustrating the implantation of A) the autograft and B) the APNF-NGC in the injured sciatic nerve.

**
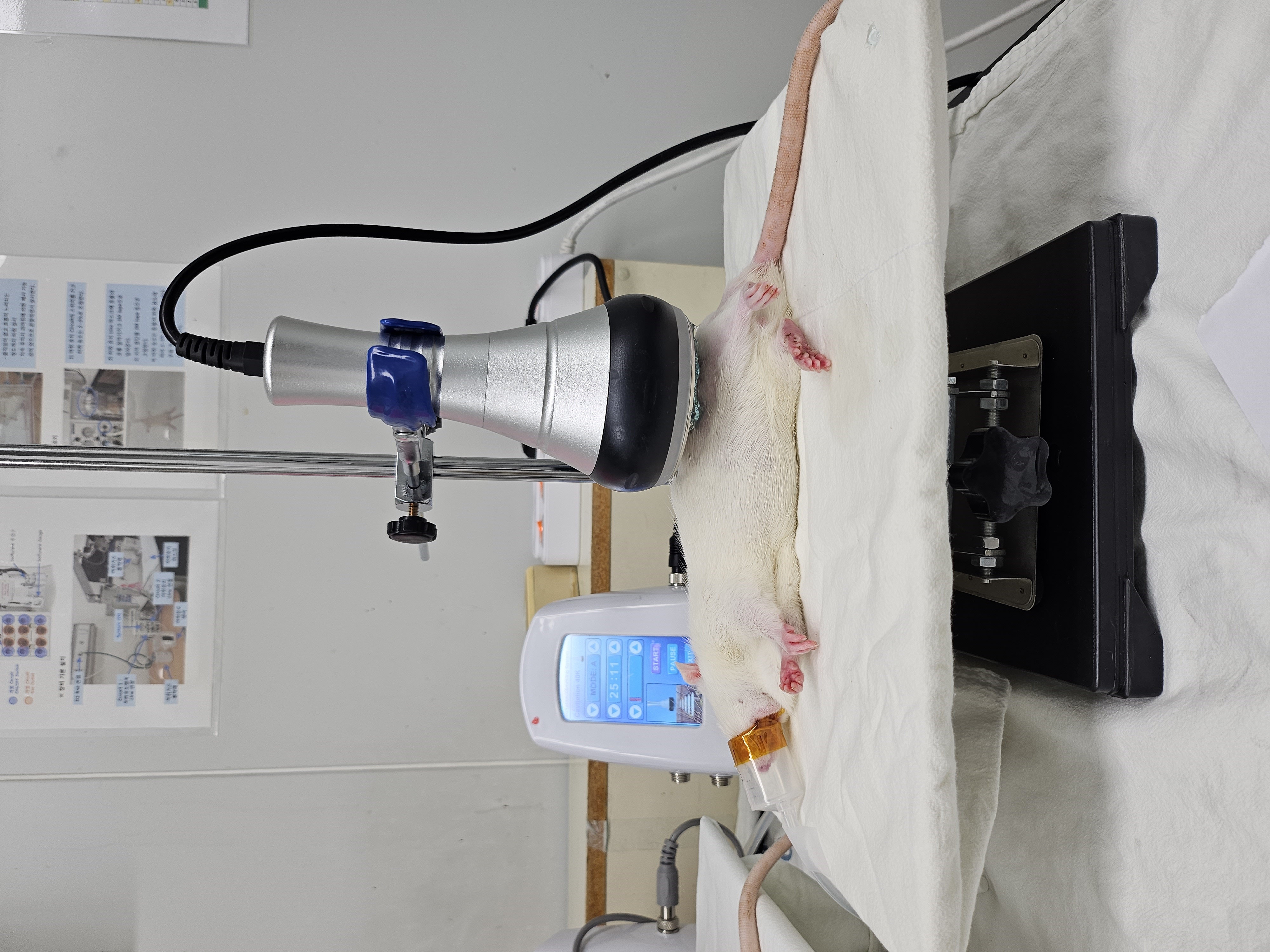
**

**Figure S15.** Photograph depicting the application of 40 kHz ultrasound to the APNF-NGC group during in vivo test. The SD rat is under anesthesia via inhalation of isoflurane (2-3% in O_2_), maintained with a nose cone.

**
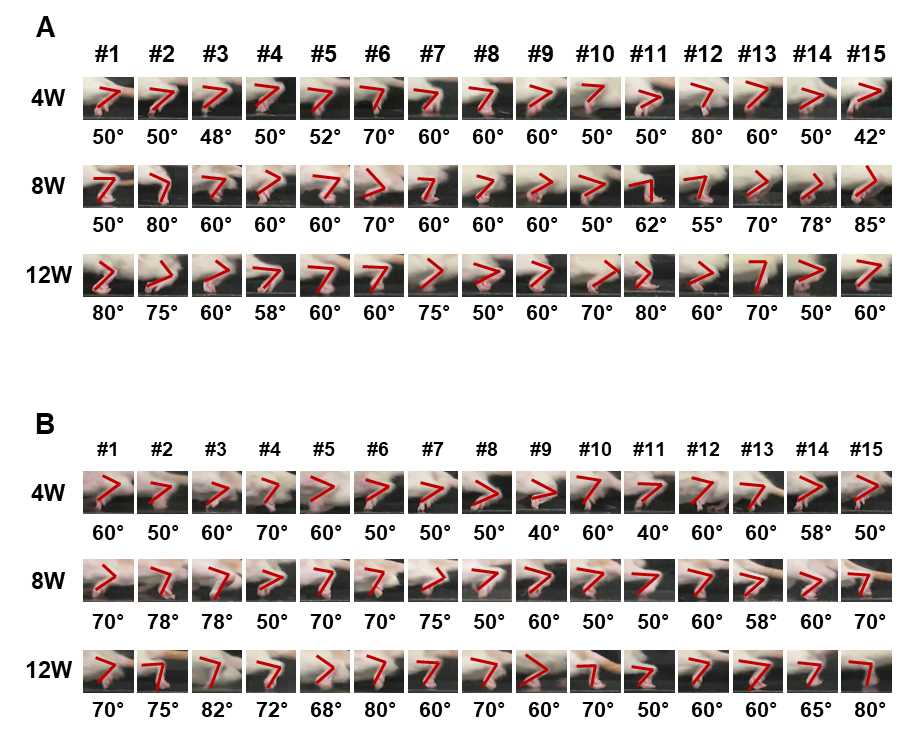
**

**Figure S16.** Captured video images from gait analysis used to measure the ankle angle during the toe-off phase, assessing limb function recovery in A) Autograft and B) APNF-NGC groups. Videos were recorded every 4 weeks post-surgery to monitor functional recovery.


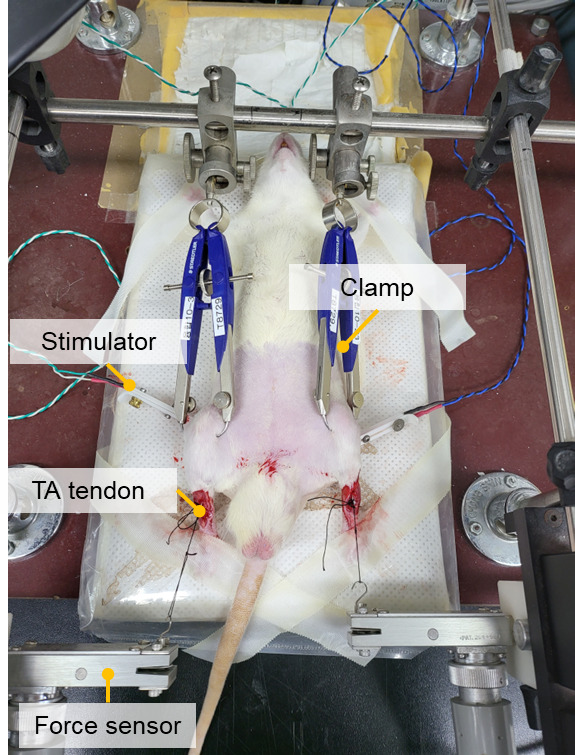


**Figure S17.** The photograph of the setup for measuring isometric tetanic force to evaluate the motor recovery.

**
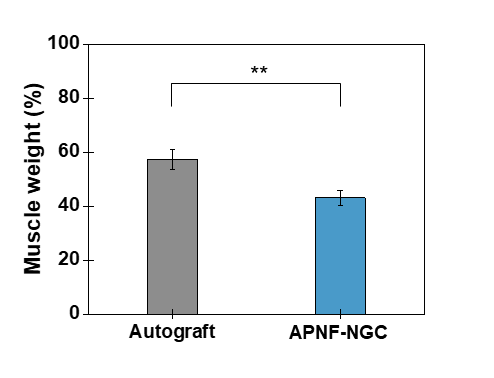
**

**Figure S18.** Quantitative comparison of tibialis anterior (TA) muscle weight between Autograft and APNF-NGC groups (values are mean ± SEM; **p < 0.01; n = 15; Student’s t-test).

**
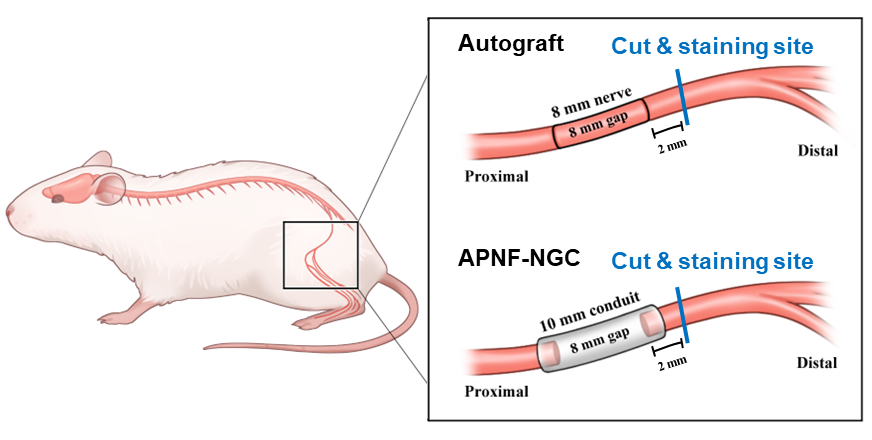
**

**Figure S19.** Schematic illustration for cutting & staining site for histologic analysis. We retrieved the transverse nerve segment from the 2-3 mm distal to the distal margin of the nerve graft.


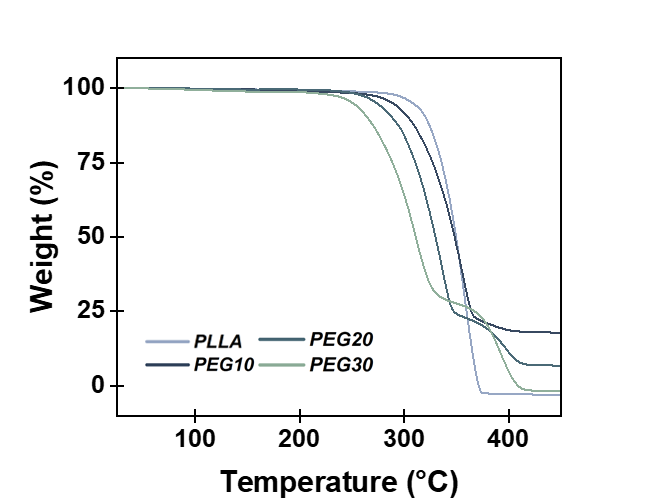


**Figure S20.** Thermogravimetric analysis (TGA) of PLLA/PEG electrospun mat with different PEG fraction.

**Table S1.** Thermal behaviors and crystallinity of PLLA/PEG nanofibers determined by differential scanning calorimetry analysis.

|  | Cold crystallization temperature  *T*_cc_ (°C) | Heat of cold crystallization  *ΔH*_cc_ (J g^-1^) | Melting temperature  *T*_m_ (°C) | Heat of fusion  *ΔH*_m_ (J g^-1^) | Crystallinity  *χ*^PLLA^ (%) |
| --- | --- | --- | --- | --- | --- |
| PLLA | 81.2 | 4.68 | 167.9 | -20.2 | 16.5 |
| PEG10 | 72.4 | 6.17 | 166.4 | -30.0 | 28.3 |
| PEG20 | 75.1 | 1.93 | 166.6 | -32.7 | 41.0 |
| PEG30 | 74.8 | 2.24 | 165.8 | -31.3 | 44.4 |

References

[1] Y. Shi, P. Kirwan, F. J. Livesey, *Nat. Protoc.*, **2012**, *7*, 1836-1846.

[2] M. W. Amoroso, G. F. Croft, D. J. Williams, S. O'Keeffe, M. A. Carrasco, A. R. Davis, L. Roybon, D. H. Oakley, T. Maniatis, C. E. Henderson, H. Wichterle, *J. Neurosci.* **2013**, *33*, 574-586.

[3] A. S. T. Smith, J. H. Kim, C. Chun, A. Gharai, H. W. Moon, E. Y. Kim, S. H. Nam, N. Ha, J. Y. Song, K. W. Chung, H. M. Doo, J. Hesson, J. Mathieu, M. Bothwell, B.-O. Choi, D.-H. Kim, *Adv. Biol.* **2022**, *6*, 2101308

[4] S. H. Yuan, J. Martin, J. Elia, J. Flippin, R. I. Paramban, M. P. Hefferan, J. G. Vidal, Y. Mu, R. L. Killian, M. A. Israel, N. Emre, S. Marsala, M. Marsala, F. H. Gage, L. S. B. Goldstein, C. T. Carson, *PloS One*, **2011**, *6*, e17540.
